# Supplementary material for: Encoding performance of cortical neurons critically depends on their morphological and neurophysiological properties
Source: PLoS Biol. 2026 May 14;24(5):e3003789. doi: 10.1371/journal.pbio.3003789 (PMC13175474; doi:10.1371/journal.pbio.3003789)
Supplement: S1 Table — (PDF) [file pbio.3003789.s001.pdf]

**Layer 5** Cell ID	$\langle I \rangle$ (pA)	σ_I (pA)	$\langle \nu \rangle$ (Hz)	**Layer 4** Cell ID	$\langle I \rangle$ (pA)	σ_I (pA)	$\langle \nu \rangle$ (Hz)
sl1_c1_030315	-147.2	304.5	7.4	sl1_c_250215	32.6	50.6	5.2
sl1_c1_250215	-32.4	253.2	5.2	sl1_c2_020315	50.4	150.1	6.7
sl1_c2_230215	95.3	354.3	10.2	sl1_c2_030315	40.7	87.4	5.7
sl1_c2_250215	-28.0	208.0	5.8	sl1_c3_020315	49.5	50.6	5.2
sl1_c2_260215	2.3	121.7	4.7	sl2_c1_020315	51.4	50.9	4.6
sl1_c3_260215	2.7	126.6	4.8	sl2_c1_230215	74.4	50.7	5.2
sl2_c1_030315	7.1	162.1	8.3	sl2_c1_260215	284.7	122.3	5.8
sl2_c2_030315	-36.1	203.2	5.9	sl2_c2_020315	55.9	50.7	4.8
sl3_c1_020315	-37.8	152.3	8.1	sl2_c3_020315	75.3	50.8	5.1
				sl3_c1_020315	97.2	51.4	5.0
	**-19.3**	**209.5**	**6.7**		**81.2**	**71.6**	**5.4**

S1 Table Stimulus details for the recordings underlying Fig. 2.
