# Supplementary material for: Encoding performance of cortical neurons critically depends on their morphological and neurophysiological properties
Source: PLoS Biol. 2026 May 14;24(5):e3003789. doi: 10.1371/journal.pbio.3003789 (PMC13175474; doi:10.1371/journal.pbio.3003789)
Supplement: S2 Table — (PDF) [file pbio.3003789.s002.pdf]

**2ms** Cell ID	$\langle I \rangle$ (pA)	σ_I (pA)	$\langle \nu \rangle$ (Hz)	**10 ms** Cell ID	$\langle I \rangle$ (pA)	σ_I (pA)	$\langle \nu \rangle$ (Hz)
sl1_c1_03_12_15	69.3	83.9	6.7	sl1_c1_03_12_15	145.0	48.5	5.9
sl1_c1_04_12_15	17.0	74.3	5.8	sl1_c1_04_12_15	21.3	46.6	6.3
sl1_c1_06_12_15	-1.3	74.1	7.3	sl1_c1_06_12_15	32.5	46.6	5.8
sl1_c2_02_12_15	-4.6	73.7	5.0	sl1_c2_02_12_15	1.4	46.3	5.1
sl1_c2_03_12_15	68.0	38.3	5.9	sl1_c2_03_12_15	67.1	23.9	6.0
sl1_c2_06_12_15	24.5	73.6	5.2	sl1_c2_06_12_15	39.0	46.6	5.0
sl1_c3_02_12_15	4.4	75.1	4.2	sl1_c3_02_12_15	14.4	46.3	4.3
sl1_c3_03_12_15	47.7	74.8	5.2	sl1_c3_03_12_15	64.5	46.8	5.6
sl1_c3_04_12_15	9.3	73.8	5.5	sl1_c3_04_12_15	12.1	46.1	5.6
sl1_c3_06_12_15	10.7	74.6	7.5	sl1_c3_06_12_15	46.6	46.3	6.8
sl1_c4_02_12_15	2.5	74.3	5.2	sl1_c4_02_12_15	8.7	46.4	5.3
sl1_c4_04_12_15	-16.3	79.3	6.0	sl1_c4_04_12_15	2.0	47.0	5.1
sl1_c5_04_12_15	115.1	73.6	5.3	sl1_c5_04_12_15	111.5	46.3	5.3
sl1_c5_06_12_15	28.4	74.8	6.2	sl1_c5_06_12_15	43.5	46.9	6.0
sl1_c6_06_12_15	28.9	74.1	5.7	sl1_c6_06_12_15	63.1	46.4	5.8
sl2_c1_03_12_15	49.7	74.1	5.0	sl2_c1_03_12_15	61.0	46.5	6.0
sl2_c1_06_12_15	20.6	74.1	6.5	sl2_c1_06_12_15	39.4	46.8	6.2
sl2_c2_02_12_15	17.3	73.8	5.3	sl2_c2_02_12_15	20.0	46.7	4.8
sl2_c2_03_12_15	2.8	74.1	7.1	sl2_c2_03_12_15	16.7	46.6	6.5
sl2_c2_06_12_15	15.8	73.8	5.5	sl2_c2_06_12_15	24.6	46.7	6.0
sl2_c3_03_12_15	20.7	73.7	5.6	sl2_c3_03_12_15	30.1	46.8	6.0
sl2_c3_06_12_15	12.9	36.8	5.7	sl2_c3_06_12_15	15.0	23.3	6.0
sl2_c4_02_12_15	43.0	73.9	4.0	sl2_c4_02_12_15	54.2	46.7	5.2
sl2_c4_03_12_15	12.5	73.8	5.5	sl2_c4_03_12_15	41.5	47.5	5.9
sl2_c4_06_12_15	26.2	37.3	5.1	sl2_c4_06_12_15	36.5	23.6	5.4
sl2_c5_02_12_15	55.2	73.8	4.5	sl2_c5_02_12_15	67.9	46.3	5.0
sl2_c5_03_12_15	-38.5	82.6	6.6	sl2_c5_03_12_15	48.8	46.5	6.2
sl2_c5_06_12_15	-2.6	74.0	6.1	sl2_c5_06_12_15	9.6	46.5	5.5
sl2_c6_03_12_15	48.4	74.2	5.4	sl2_c6_03_12_15	64.6	47.4	5.8
sl2_c7_06_12_15	-10.6	73.8	7.0	sl2_c6_06_12_15	-5.9	46.2	6.2
sl3_c2_03_12_15	41.9	37.4	4.2	sl3_c2_03_12_15	46.1	25.3	5.0
	**23.2**	**70.1**	**5.7**		**40.1**	**43.8**	**5.7**

S2 Table Stimulus details for the recordings underlying Fig. 4.
