# Supplementary figures and images for: Encoding performance of cortical neurons critically depends on their morphological and neurophysiological properties
Source: PLoS Biol. 2026 May 14;24(5):e3003789. doi: 10.1371/journal.pbio.3003789 (PMC13175474; doi:10.1371/journal.pbio.3003789)

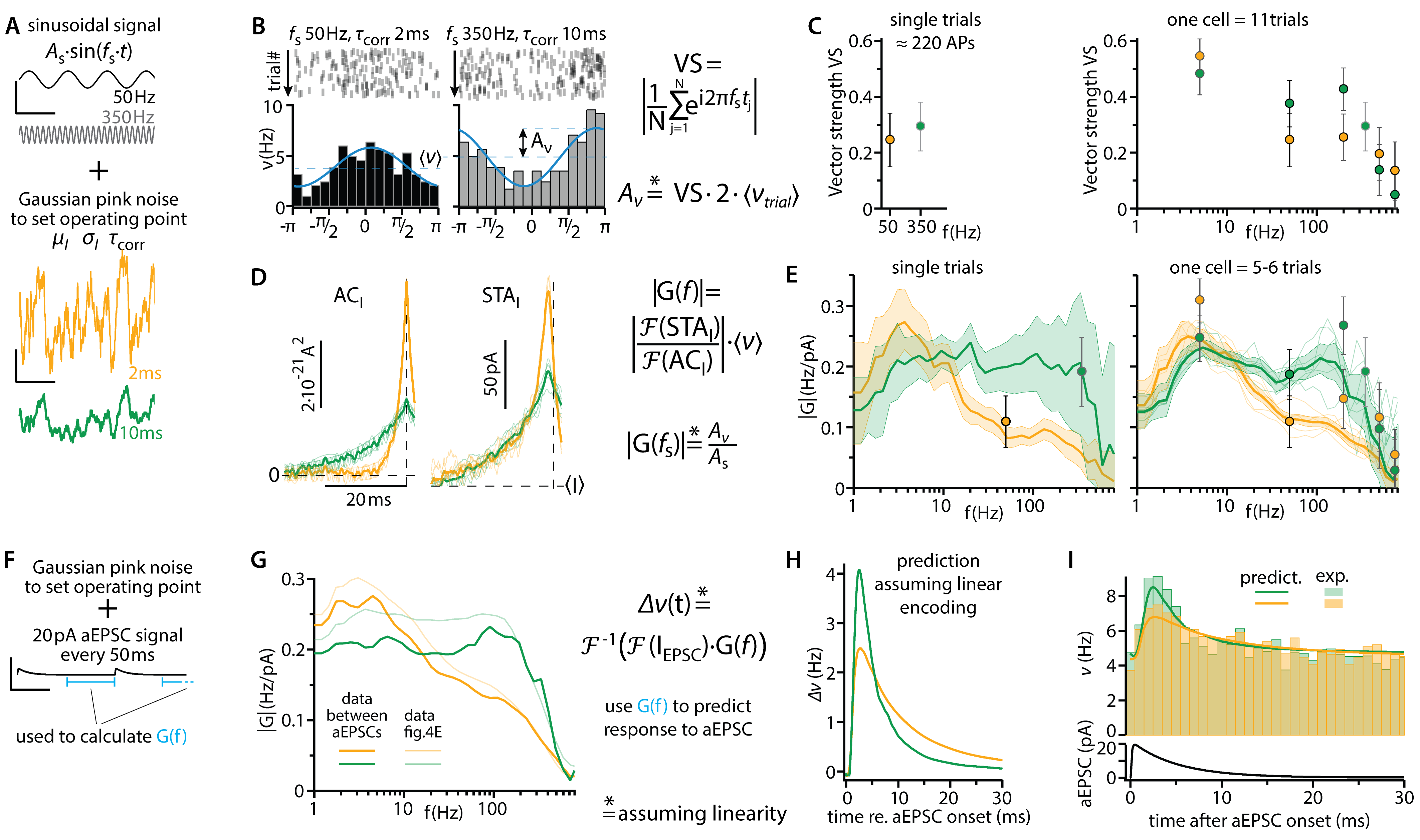

Supplement: S1 Fig — (A) In all cases, the neurons’ working points are set by fluctuating inputs with a correlation time of either 2 ms (orange) or 10 ms (green). Mean and standard deviation are chosen to achieve irregular firing with an average rate 〈ν〉 around 5 Hz. (see Fig 4A). This stochastic background stimulus (Ornstein–Uhlenbeck-Type, see Materials and methods), can be combined with a periodic, sinusoidal input. Here, examples are shown with frequencies fs of 50 Hz (black) and 350 Hz (gray). (B) When AP times are evaluated only with respect to the phase of the added sine component, the sine-locked firing rate is obtained (bars). It follows the sinusoidal input modulation with a phase shift and a modulation depth amplitude Aν. Each of the two panels displays data of a single, 45-second-long stimulation trial, evoking about 220 APs. (C) From all AP times tj the vector strength can be calculated. The results for the two example trials from B are shown in the left panel. The right panel contains the results for all 11 trials in this cell (5 different sine frequencies for 2 ms correlated background and 6 frequencies for 10 ms). Shown are average and 95% confidence intervals. (D) AP times can also be evaluated with respect to all the frequency components that jointly constitute the noisy background and the sine component. To do so, the input auto-correlation ACI and the spike-triggered average input STAI were obtained from the total input and the times tj, at which action potentials occurred. Shown are results for the 11 individual trials (semi-transparent) and for the average of the trials with the same correlation time (opaque). Note that the ACI of each trial clearly displays a periodic component, reflecting the sinusoidal current that had been added to the stimulus (see A). (E) The dynamic gain function G(f) can be calculated as the ratio of the Fourier-transformations of STAI and ACI, multiplied with the average firing rate. The left panel shows its magnitude |G| for two individua [file pbio.3003789.s003.tif]

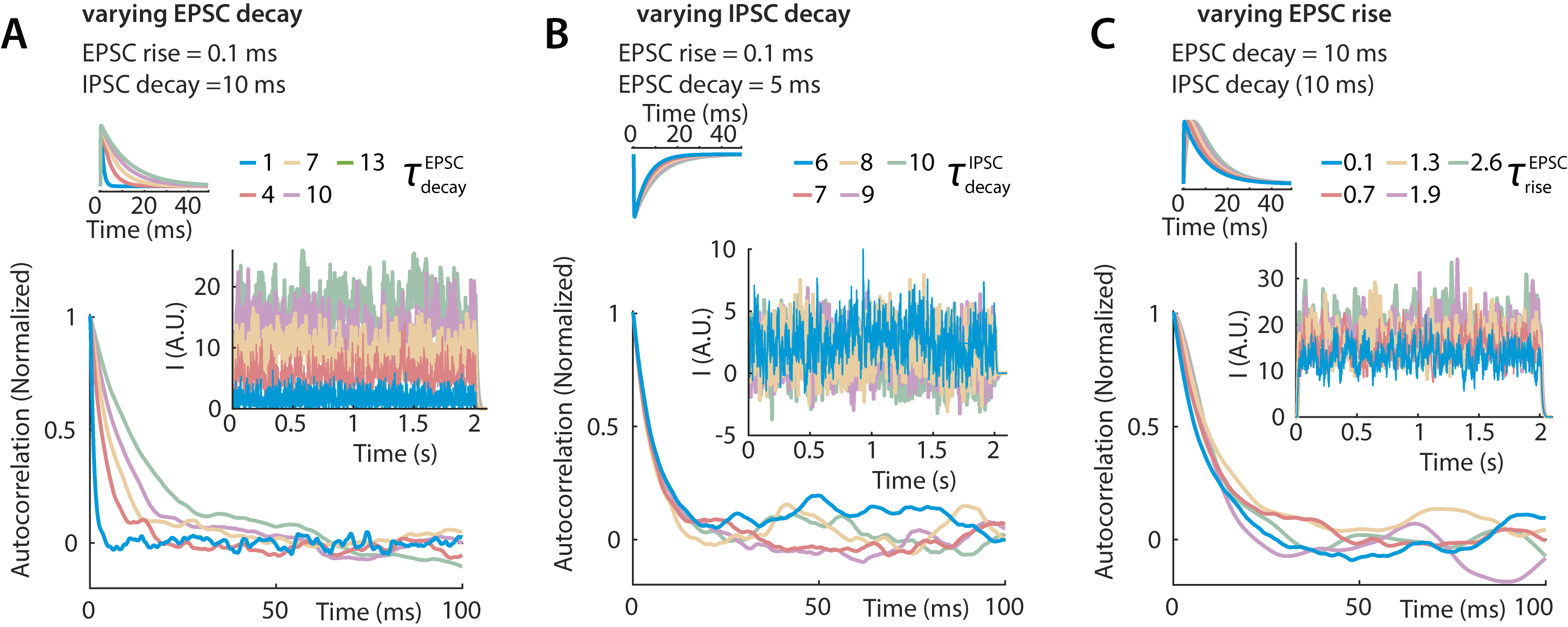

Supplement: S2 Fig — In the model, the frequencies and amplitudes of the excitatory and inhibitory synaptic conductances are the same. (A) It can be seen that, as the decay time constant of the EPSCs increases, the time correlation of the noise is markedly slowed. (B) By contrast, similar changes in the decay of the inhibitory synapses have a much smaller effect on the characteristics of the noise because the driving force for inhibition is quite small at −50 mV. (C) Similarly, the noise characteristics is also not very sensitive to the rise time of the EPSCs (but see the minor widening of the autocorrelation at its peak). (TIF) [file pbio.3003789.s004.tif]

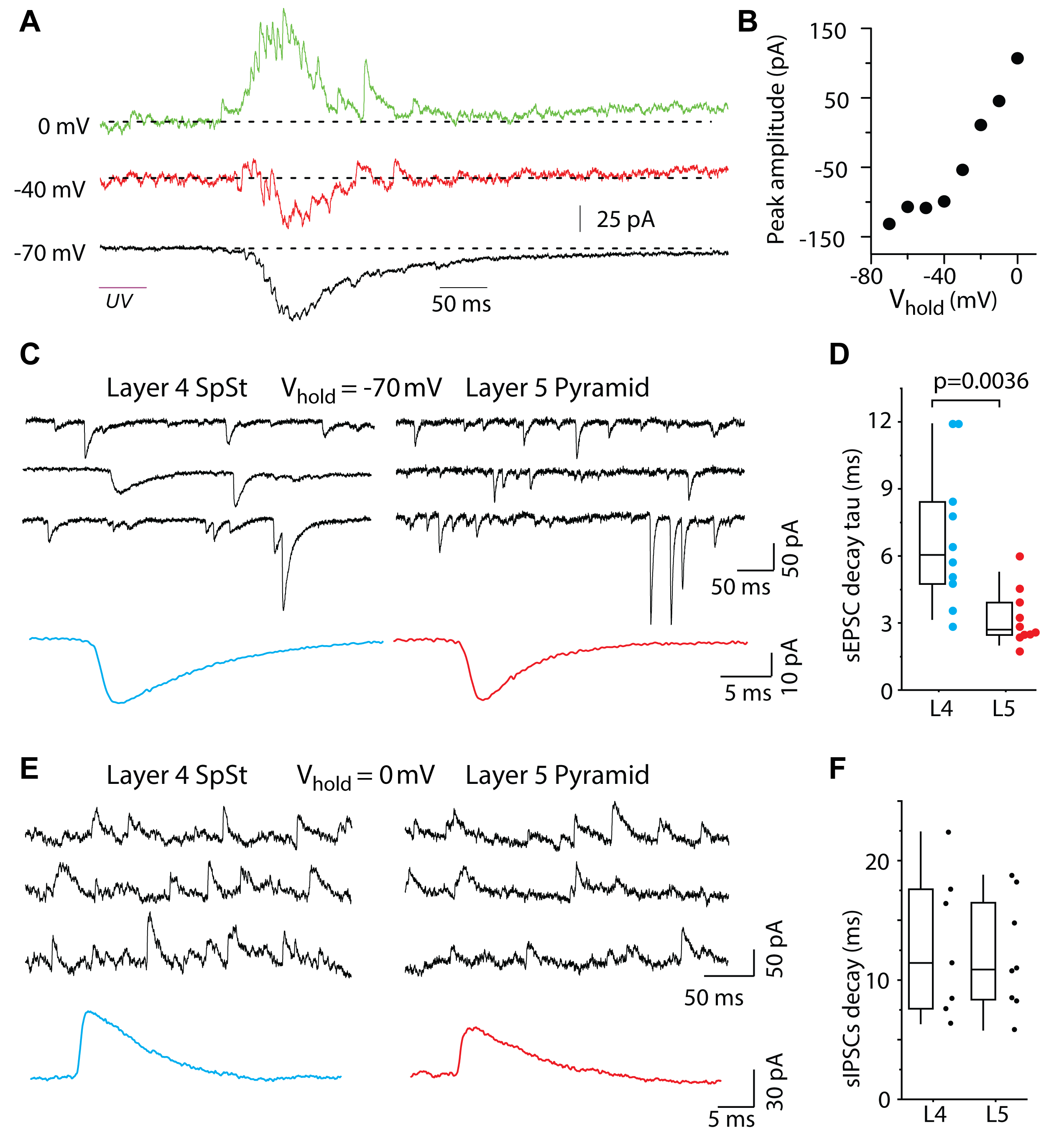

Supplement: S3 Fig — Data DOI for (B),(D), and (F): https://doi.org/10.25625/VQUKFU. (TIF) [file pbio.3003789.s005.tif]
