# Supplementary material for: Encoding performance of cortical neurons critically depends on their morphological and neurophysiological properties
Source: PLoS Biol. 2026 May 14;24(5):e3003789. doi: 10.1371/journal.pbio.3003789 (PMC13175474; doi:10.1371/journal.pbio.3003789)
Supplement: Attachment — Submitted filename: R1.pdf [file pbio.3003789.s006.pdf]

This manuscript by Revah et al. investigates how the temporal fidelity of cortical population coding arises from specific cellular and synaptic properties. Using dynamic gain analysis, the frequency-dependent linear response of a neuron or population to input fluctuations, the authors combine theoretical modeling with electrophysiological experiments in rodent barrel cortex. They focus on Layer 4 (L4) spiny stellate cells as a "stable, defined population" receiving known thalamic input. The study tests predictions that a population's encoding speed depends on cell number, cell size, and background noise correlation time Key findings are that L4 neurons indeed exhibit a broad high-frequency bandwidth and high response fidelity, enabled by their small size and unusually long synaptic noise correlation time. These factors appear "fine-tuned" together with the number of L4 neurons such that even a single thalamocortical spike in the input can be reliably detected in the L4 population output. Additionally, pharmacological manipulation of K_V7 (M-type) potassium channels shows that M-current activity modulates the speed of action potential initiation, thereby adjusting the dynamic gain. This suggests that the fast relay function of L4 can be gated by cholinergic (muscarinic) brain states. Overall, the authors provide a mechanistic link between cellular physiology (e.g. spike initiation kinetics, synaptic kinetics) and the sub-millisecond precision of population coding in cortex. The questions addressed are highly significant for understanding cortical information processing. While it has been known that cortical neurons can encode surprisingly high-frequency input changes, this work pinpoints how specific biophysical parameters are matched to achieve near-optimal encoding in an actual cortical microcircuit.

While the study is compelling, a few weaknesses or points of clarification should be addressed:

1) A potential concern is how well the slice experiment results translate to the intact, awake brain. In vivo, cortical neurons receive continuous barrage from ongoing activity, and neuromodulatory tone differs across brain states. The authors should clarify the assumptions made when extrapolating from their in vitro "stable population" to in vivo function. For instance, if the slice was in an artificial down-state (with very low spontaneous activity except what was injected), how might an up-state or aroused condition (with higher baseline synaptic noise) affect the dynamic gain? They partially address this by discussing muscarinic modulation, but it would strengthen the paper to explicitly note that in awake conditions L4 might already be in a high-conductance state – and to speculate how that interacts with their findings. In particular, would the "single thalamic spike → output" rule still hold amidst ongoing cortical activity? If possible, the authors could discuss evidence from in vivo studies (e.g. Bruno & Sakmann 2006 showing reliable L4 spiking to single thalamic events) to support that their slice results reflect real function.

2) The claim that even one thalamocortical spike is reflected in population output is exciting but could be interpreted in different ways. Does it mean that one presynaptic spike in one VB thalamic neuron can trigger at least one spike in L4 (across the population) on essentially every occurrence? This would imply an extremely high sensitivity. Classical studies suggest thalamic inputs are weak individually, and a few synchronous thalamic spikes are needed to drive an L4 cell to fire. The authors should clarify this point. It may be that within a barrel, because there are ~100 spiny stellates receiving partially redundant thalamic afferents, the population as a whole fires at least one spike for each thalamic spike (even if any given single neuron is probabilistic). If that is the interpretation, the authors might explicitly state it to avoid confusion. As a suggestion, providing a quantitative estimate of L4 population spike probability per thalamic input (and how that compares to chance) would help substantiate the "reliable relay" claim. If this is based on modeling rather than direct measurement, that should be stated. In any case, a clearer explanation would strengthen the reader's confidence that this is not an overstatement.

3) The finding that L4's background noise has a longer correlation time than other layers is intriguing. The manuscript would benefit from a more detailed discussion of why this is the case biophysically. Do L4 cells have different receptor compositions or network connectivity that prolong synaptic events? For example, is it due to a higher NMDA/AMPA ratio, or more GABA_B involvement, or simply that most "noise" in L4 comes from recurrent activity with longer integrative times? Citing specific data (perhaps from their recordings of synaptic kinetics, or known receptor differences) would support this point. In the text, they mention "synaptic receptor dynamics" as the cause. It would help readers if the authors specify which receptors dominate L4 noise, e.g. "slow NMDA-mediated EPSPs and GABAergic currents lengthen the autocorrelation of L4 synaptic input." This also ties into literature: slow glutamate receptors can sustain depolarizations and thus increase the integration window. A brief comparison to layers 2/3 or 5 (which likely have faster AMPA-dominated transients) could contextualize the uniqueness of L4.

4) The demonstration with K_V7 channels is convincing, but a few clarifications are warranted. K_V7 (M-type) channels are one of several subthreshold conductances (others include HCN/I_h and persistent Na^+). The authors focus on K_V7 likely because their effect was strong, but it would be useful to acknowledge whether other channels were considered or could play a role. For instance, I_h currents can also influence responsiveness by depolarizing neurons and speeding up rebound firing. Did the authors assume I_h was negligible in these L4 cells or kept constant? A sentence discussing this would show thoroughness. Additionally, XE991 (the M-channel blocker) can have off-target effects at high concentrations; the authors should note the concentration used and any steps taken to ensure changes in gain were indeed due to M-current block (e.g. reversibility, known effects on input resistance, etc.). Since the manuscript's main claim here is that M-currents modulate spike initiation speed, they might consider directly correlating the AP onset rapidness (slope of rise to threshold) before and after M-channel block. This would link to prior work showing that AP onset dynamics govern high-frequency encoding. Providing such a correlation (if available) would further strengthen the mechanistic point.

5) The authors repeatedly use terms like "optimized" and "fine-tuned." While they do demonstrate that L4 meets certain theoretical predictions, it would be beneficial to see how sensitive the encoding performance is to deviations in those parameters. For example, if L4 cell soma size were 20% larger, or if synaptic time constants were 50% faster, does the high-frequency cutoff drop precipitously? The manuscript would benefit from either data or discussion on this point – essentially, an analysis of robustness. Such information would convey how precisely tuned the system is and could reveal which factor is most critical. It also helps readers appreciate whether evolution had to precisely adjust all factors or if there's some tolerance. If the authors did not quantitatively do this, they should at least qualitatively discuss it, given it's central to their claim of fine-tuning.

6) In the Discussion, the authors should amplify the implications of their findings for sensory processing and behavior. They touch on this (e.g. mentioning that coding in L4 may vary with brain state, but they could go further. For instance, the fact that L4 can reliably transmit single spikes suggests the cortex can operate on a single-spike per neuron basis for initial sensory detection. This resonates with theories of fast feedforward processing in vision and touch, where very few spikes are needed for perception. The authors might speculate on how the fine-tuning in L4 could be a general principle in other sensory cortices, or discuss if similar optimization might be found in auditory or visual L4 (noting differences, e.g. auditory cortex lacks a discrete L4 in rodents. Additionally, since they link to muscarinic control, they could mention potential behavioral scenarios: during attentive whisking, cholinergic tone is high, which might transiently enhance high-frequency transmission in barrel cortex, possibly improving detection of rapid whisker vibrations or

changes in texture. Making these connections would strengthen the significance of the work for a broad neuroscience audience.
