# Supplementary material for: Encoding performance of cortical neurons critically depends on their morphological and neurophysiological properties
Source: PLoS Biol. 2026 May 14;24(5):e3003789. doi: 10.1371/journal.pbio.3003789 (PMC13175474; doi:10.1371/journal.pbio.3003789)
Supplement: Attachment — Submitted filename: PLOS-BIOL-V2-ref.pdf [file pbio.3003789.s008.pdf]

The authors have improved the ms but I am not satisfied with some of the replies and the corresponding revisions. For some of the issues I raised, my remarks were possibly too brief, so I elaborate on some of them below. For my final recommendation it is essential that the authors seriously address all of them.

Major issues

1) The authors study the role of the correlation time of the background fluctuations, however, as the source of correlation they name only synaptic filtering. In the theoretical literature, one additional potential source of correlation are the recurrent activity itself, that, specifically at larger synaptic amplitudes, can give raise to long correlations exceeding those introduced by synaptic filter times, see e.g. Ostojic Nature Neurosci 2014. The authors make a point that in their in vitro recordings correlation times match the synaptic decay times, but they should still discuss this second (more complicated) source of correlations and argue why - or, better, under which conditions - the synaptic filtering will dominate the correlation time.

Thank you for the suggestion. We added a paragraph to the discussion:

"An alternative mechanism for slow input fluctuations could be a slow oscillation of the recurrent network itself. Although this would assure conditions for a rapid response of L4 neurons without the need for special synaptic receptors, those oscillations would pose a serious problem for L4 function because it would create prolonged periods during which most neurons were far from threshold and the population's input sensitivity would be reduced. Indeed, in-vivo recordings from mouse S1 show that during active sensing periods, the networks in S1 switch to asynchronous activity[31], thereby avoiding intermittent drop-outs. The input correlation time is at least as long as the decay time constant of the dominant postsynaptic currents, even if the network activity is completely asynchronous. The presence of slow synapses, thereby, allows L4 neurons to consistently respond rapidly."

I am afraid I did not make myself sufficiently clear. I did not refer to a network oscillation but to the correlation of background noise that arises from the fact that the underlying superposition of spike trains from the other (the presynaptic) neurons is in general correlated. The synaptic filter, exclusively used by the authors as the source of effective noise correlations, would be meaningful only, if the neuron were driven by white (say, Poissonian) noise. However, the superposition of presynaptic spike trains that constitute the input to the synaptic filter is in most cases already correlated because neural spiking is correlated (correlations do not vanish by superposition as one might naively think, see Lindner Phys. Rev. E 2006).

The hallmark of the temporal correlations of this recurrent input is that it is related to the spike train statistics of the driven cell. For a statistically homogeneous network this can be found (in theory) from a self-consistency condition connecting the correlation function of the input noise to the correlation function of the neuron under consideration. This has been investigated for a network of rate units, e.g. in the famous paper by Sompolinsky, Crisanti, and Sommer Phys. Rev. Lett. 1988, or for spiking neurons by Lerchner et al. Neural Comput. (2006). The correlation times caused by the nonlinear interactions in the network can easily become larger than those induced by the synaptic filter, see, for instance, Wieland et al Phys. Rev. E (2015), specifically, if synaptic amplitudes in the network are large (Ostojic, Nat Neurosci 2014).

It would be good if the authors had a look at these papers and commented whether network-interaction-induced correlations may also play a role in the considered system. One indication for long correlation times in the background noise (before the synaptic filter stage!) would be that a substantial fraction of neurons displays a coefficient of variation (CV) of the interspike interval that is larger than one. Put differently, if the spike train statistics of the neurons that generate the background noise is Poisson-like or even more regular (CV equal one or smaller than one, respectively), one can exclude that the network noise contributes significantly to the color of the background noise. The latter would then be indeed dominated by the synaptic filter as assumed by the authors.

2) On p.2 the authors write:

"In cortical networks, the postsynaptic effect of local, recurrent inputs reflects a balance of inhibition and excitation, and it is largely asynchronous from neuron to neuron."

This might be true but the weak remaining cross-correlations can have a strong impact on the population activity (see the famous contribution on this problem by the Bialek group, Schneidman et al. Nature 2006). So it would be important to state how strongly the L4 neurons are correlated exactly. And one could take into account how weakly correlated noise sources to different L4 neurons could mimic this effect. At least this problem should be mentioned and the limitations of the current approach (based solely on single-cell activity) should be pointed out.

To our knowledge, information on the correlation between L4 spiny stellates are not yet reported. We now mention the limitation of the current approach:"

"Because in vivo a thalamocortical volley is immediately followed by strong feedforward and feedback inhibition 37,38, our data indicate that the slower correlation time of the noise is essential for the fast encoding that is actually achieved by L4. Note that in spiny stellate cells in vivo, the background noise largely reflects recurrent activity, and the histogram bars after the initial response might be modified as a consequence of the thalamocortical EPSC. However, only the first one or two bars are relevant for our conclusion (Fig. 5D)."

I do not see how the authors have discussed or even only mentioned the contribution of cross correlations among the neurons (not to be confused with the temporal correlations of the noise or the spike train) for the considered problem. If neurons are not completely independent of each other, i) the population rate would be much more noisy than for independent neurons and ii) also the presynaptic input would be shaped by this. This may affect some of the arguments made in the context of fine tuning of population size, cell size, and correlation time of the noise. The authors' approach assumes complete independence (a perfectly asynchronous state); please, comment on the limitations of these conclusions due to possible violations of this assumption.

3) Statements about the DC, AC, and noise injections should be completed with a table of the used values.

The authors write on p.15

"We adjusted the input's standard deviation, σI, based on the input resistance of the neuron, to assure that the voltage fluctuations were similar in all neurons. Along with σI, we also scaled the amplitude of the sinusoidal component $Asin=0.37 \cdot \sigma I$. A constant, direct current (DC) $\langle \rangle$ was added and adjusted to maintain a target firing rate of ~5 Hz."

This is not reproducable, because we do not know the SD of voltage fluctuations. Different SDs could in combination with different DC currents result in the same 5 Hz firing rate. I suggest to give all the values in a table in the supplementary material: neuron number, correlation time and SD of OUP input current, amplitude of sinusoidal component, DC current, measured mean firing rate, measured maximal amplitude of firing rate modulation. In this way the reader (and in the first place, this reviewer) would get an idea about i) the variability of cells, ii) the susceptibility of the voltage variance with respect to the noise amplitude, iii) the (non)linearity of the signal transmission.

The question of linearity is central to the dynamic gain approach, which is why we explicitly tested for linearity. Specifically, we compared the experimentally measured EPSC response with the response we predicted under the assumption of linearity. The result strongly supports the assumption of linearity for the EPSC response. This is shown in Fig. S1 "Linear ansatz to quantify encoding is confirmed by comparison of three different approaches" (previously Fig S3)

The assessment of linearity through comparison of average firing rate $\langle \nu \rangle$ and sine-modulation of the rate A_ν is also available in the manuscript, by way of the vector strength (VS). This can be appreciated from the equation in Fig S1:

$\langle \nu \rangle / A_\nu = 2VS$.

The vector strength values for the individual trials are shown in Fig. 4 of the manuscript. We show it here next to the vector strength values for the individual trials that constitute Fig. 2:

The tables with the other requested information are now included in the Supplementary materials and reproduced here:

I am happy with the revision regarding this point. Thank you for adding the tables.

4) The authors compare the stimulation by a (comparatively) weak periodic signal and the estimation of the linear response function from the vector strength to the estimate of the response function from the full input-output cross-spectrum between the (non-weak) Gaussian noise stimulus and the output spike train. That these functions are the same (at least for an sufficiently weak cosine stimulus) is, according to my understanding, a consequence of the Furutsu-Novikov theorem that has been recently (re)derived in the context of spiking neurons by Lindner Phys. Rev. Lett. 2023. This connection should be mentioned.

Thank you for the reference. We now incorporated it in the text box on Dynamic gain:

"An alternative method to derive the dynamic gain function does not require an additional sinusoidal input (see Methods: Dynamic gain calculation and Fig. S1D to S1E) because the stochastically fluctuating input is, itself, composed of various frequency components. The results of these two approaches are not only theoretically identical (Stubenrauch and Lindner 2024) but also identical in our experiments (Fig. S1E)."

The given reference is not correct: Stubenrauch and Lindner derived a Furutsu-Novikov-like theorem for Poissonian spike trains with rate modulation – this is not relevant, I think, for what is done in the submitted paper. The correct (original) theorem applies for Gaussian signals (as used by the authors); it is the one rederived in the paper by Lindner that I mentioned in my first report. I do not understand why the authors cite here the other paper.

5) Why the cell with larger dendrite has a broader bandwidth of transmission has been discussed at length in the paper by Ostojic et al. J Neurosci (2015). The paper is already cited in the paper but the mechanism should be briefly explained because the difference in bandwidth between layer IV and V cells is such an important starting point of the paper. Additional references that might be useful and merit citation in this context are those by Gowers and Richardson Phys. Rev. Res. (2023) and Gowers, Timofeeva, and Richardson PLoS Comput. Biol. (2020) who studied analytically response functions in spatially extended neuron models.

We added a sentence and a reference to our earlier analysis of different effects of dendrites on encoding bandwidth (Zhang, C., Revah, O., Wolf, F., and Neef, A. (2024). Dynamic gain decomposition reveals functional effects of dendrites, ion channels and input statistics in population coding.)

"These critical features include the correlation time of the background noise (Brunel et al. 2001), the dynamics of action potential generation (Brunel et al. 2001, Naundorf et al. 2005), and the dendritic size of the neurons involved (Ostojic et al. 2015, Eyal et al. 2014). Dendrites amplify the impact of rapid input fluctuations because they shunt more slowly changing inputs (Ostojic et al. 2015, Zhang et al. 2024)."

While we appreciate the additional references, those studies are more related to the encoding of dendritic inputs, and increased dendritic conductances. We felt that for the topic of our study, it would be fair not to include those references because the inputs onto the spiny stellate cell are all electrotonically close to the soma (Segev I, Friedman A, White EL, Gutnick MJ. Electrical consequences of spine dimensions in a model of a cortical spiny stellate cell completely reconstructed from serial thin sections. J Comput Neurosci. 1995;2: 117–130. doi:10.1007/BF00961883)

I understand this argument but do not see, why the authors do not share it with the readers. I recommend to briefly mention the effect of the dendritic tree on transmission band width (and cite the

above references) and then state why this is not relevant for the spiny stellate cells (citing the paper mentioned in the reply).

Minor issues

6)p.16
"Fourier transform of the autocorrelation" This is well-known as the power spectrum!

In our theory-oriented papers, we indeed make that transition via the Wiener-Khinchin theorem. However, I asked a number of non-computational experimentalists whether they were aware that the power spectrum is, in fact, the Fourier transform of the autocorrelation. Our wording reflects our deference to the broad readership of PLoS Biology.

I do not understand why the lack of knowledge in a certain group of researchers keeps the authors from adding a simple parenthesis “(also known as power spectrum)” to mention an important term in the theory of stochastic signals. I highly recommend to add it.
