# Supplementary material for: Encoding performance of cortical neurons critically depends on their morphological and neurophysiological properties
Source: PLoS Biol. 2026 May 14;24(5):e3003789. doi: 10.1371/journal.pbio.3003789 (PMC13175474; doi:10.1371/journal.pbio.3003789)
Supplement: Attachment — Submitted filename: Response to the review R2.pdf [file pbio.3003789.s009.pdf]

Reviews PBIOLOGY-D-25-02148R2

Text with grey background originates from the first review round and is here, only where the reviewer had chosen to keep it in.
Blue Reviewer comment from second round
Red Author response
Black Quotes from the manuscript that are unchanged. They are given to provide context and save the reviewers and editors looking up the corresponding manuscript page.

Reviewer #1:

The authors thoroughly address all of my more minor general comments; however, the problem remains that the title and abstract of the paper do not accurately reflect their work. Regardless of the fact that the first 1-2 ms reflect primarily feed-forward inputs (a statement with which I have no argument), the authors ignore the significant connotations of the term "cortical population," with its related implications. Your title "Cortical population coding critically depends on fine-tuning of cell physiology" does not reflect that those implications are violated in this study. It well established that thalamic inputs constitute a minor fraction of cortical encoding of stimuli (Lien 2013), so considering subsequent cortical interactions is usually implicit in a paper titled "Cortical population coding….." If you are not addressing this point, the title and abstract should be modified to very clearly state the context you are considering: Early cortical responses to feed-forward thalamic inputs. Nowhere in the abstract is this limitation stated.

We hope that the following change in the title accommodates your concerns:

from

> "Cortical population coding critically depends on fine-tuning of cell physiology"

to

"Coding in cortical subpopulations critically depends on fine-tuning of cell physiology"

Concerning the scope of our findings, we implied in the introduction that the encoding problem is universal, but that a quantitative experimental investigation is only possible in certain regions.

> "Experimental assessment of these theoretical concepts requires a stable population of well-defined neurons, in which each of the above factors is known. However, a cortical neuron may participate in many functional networks, and at any moment, a relevant sub-popu-lation of neurons is sparsely embedded within a larger network (Fig. 1A). Moreover, the characteristics of the common input are usually unknown. Here, we overcome these problems by studying the encoding performance of a stable population of well-described cortical neurons: the excitatory cells of Layer 4 (L4) of the mouse somatosensory cortex "

We have now added a sentence to the discussion (below in red) regarding the possible scope of the approach and its results.

Fundamentally, cortical function entails the passage of information between neuronal ensembles composed of neurons whose output collectively represents common input at a given point in time. However, a given neuron joins different populations at different moments, and the specific function that is served is usually not known. By studying this encoding in a well-defined subpopulation with known inputs, we identified principles of encoding that may well apply to many other cortical areas.

Reviewer #2:

The authors have improved the ms but I am not satisfied with some of the replies and the corresponding revisions. For some of the issues I raised, my remarks were possibly too brief, so I elaborate on some of them below. For my final recommendation it is essential that the authors seriously address all of them.

We understand your points better, now that you explained them further and we address them below. This said, ours is an experimental study. It is based on prior theoretical studies that predicted that small neurons, such as the spiny stellate cells of layer 4, should have slow responses unless the background input is slowly correlated. So from the start, we were aware of the importance of the input's temporal statistics. This realization made us perform the first-ever *measurements* of the dynamics of background current noise in cortical neurons. Because we measured it, we can be confident that our results are obtained under realistic input statistics.

In this respect, please allow us to stress that we highly appreciate this referee's emphasis on the multifaceted issues raised in the theoretical literature on the origin of temporally correlated inputs to cortical populations, even in asynchronous and weakly synchronous states. We had expanded the coverage of these aspects in the discussion of the present manuscript and revisited this section to make sure we avoid irritations that might distract the reader from the subject of the current paper's experimental results, that is, the phenomena and theoretical predictions pertinent to the population in the barrel cortex Layer 4.

Before addressing your comments in detail, we would like to summarize the essence of why such aspects of temporal and spatial correlations are not pertinent to the validity or conclusions of the current paper and are thus properly treated in the discussion section. You were pointing out that, besides synaptic receptor and other postsynaptic time constants, serial spike correlations in the afferent spike trains are expected to contribute to the complete correlation function (Lerchner et al. 2006). As a second important point, you highlighted that in statistically heterogeneous ensembles (such as retinal ganglion cell populations of diverse types and receptive fields) weak pairwise correlations can go together with probabilities of concerted firing patterns that are way bigger than conditional independence of responses would predict (Schneidmann et al. 2010).

Exactly because we recognize the fundamental importance of these insights for understanding more complex cortical populations, we are convinced that the population of barrel cortex L4, investigated in our study, is exceptionally suited to test the predictions on the dynamics, speed and sensitivity of coding by uniform populations derived in the seminal studies of Brunel (Brunel et al. 2001) and Eyal (Eyal et al. 2014). We agree with your judgement that none of these

predictions depended on the precise origin of temporal current correlations, and we recall that none of them were called into question in the theoretical literature. Unlike the populations Schneidmann and the maximum entropy community are focusing on, our L4 population is as uniform in composition and input structure as it gets. In addition, in this uniform population, we are examining a short transient response from a low-firing-rate spontaneous activity state. A potential contribution of serial correlations (Lindner 2001, Lerchner et al. 2006) will concern timescales on the order of the inverse firing rate, which in our study would be between 200ms and 1s. In our current recordings, such long timescales are not evident, and thus we find it likely that their impact is, in fact, weak. We are confident that, in light of these general considerations, the reviewer will agree with us that these issues are in all likelihood of minimal relevance to the conclusions of the current study, and it is proper to treat these interesting questions in the discussion section.

Major issues

1) The authors study the role of the correlation time of the background fluctuations, however, as the source of correlation they name only synaptic filtering. In the theoretical literature, one additional potential source of correlation are the recurrent activity itself, that, specifically at larger synaptic amplitudes, can give raise to long correlations exceeding those introduced by synaptic filter times, see

e.g. Ostojic Nature Neurosci 2014. The authors make a point that in their in vitro recordings correlation times match the synaptic decay times, but they should still discuss this second (more complicated) source of correlations and argue why - or, better, under which conditions - the synaptic filtering will dominate the correlation time.

Thank you for the suggestion. We added a paragraph to the discussion:

"An alternative mechanism for slow input fluctuations could be a slow oscillation of the recurrent network itself. Although this would assure conditions for a rapid response of L4 neurons without the need for special synaptic receptors, those oscillations would pose a serious problem for L4 function because it would create prolonged periods during which most neurons were far from threshold and the population's input sensitivity would be reduced. Indeed, in-vivo recordings from mouse S1 show that during active sensing periods, the networks in S1 switch to asynchronous activity, thereby avoiding intermittent drop-outs. The input correlation time is at least as long as the decay time constant of the dominant postsynaptic currents, even if the network activity is completely asynchronous. The presence of slow synapses, thereby, allows L4 neurons to consistently respond rapidly."

I am afraid I did not make myself sufficiently clear. I did not refer to a network oscillation but to the correlation of background noise that arises from the fact that the underlying superposition of spike trains from the other (the presynaptic) neurons is in general correlated. The synaptic filter, exclusively used by the authors as the source of effective noise correlations, would be meaningful only, if the neuron were driven by white (say, Poissonian) noise. However, the superposition of presynaptic spike trains that constitute the input to the synaptic filter is in most

cases already correlated because neural spiking is correlated (correlations do not vanish by superposition as one might naively think, see Lindner Phys. Rev. E 2006).

It is not correct, that we exclusively used synaptic filters as the source of effective noise correlations. We used a correlation time of 10 ms, longer than the observed 7 ms average correlation time. We now specifically state this in the results.

> For the faster correlation time, we chose 2 ms, which is at the lower end of the correlation times measured in L5 pyramidal cells. For the longer correlation time, we used 10 ms, slightly longer than the average 7 ms observed in L4 network reverberations.

The hallmark of the temporal correlations of this recurrent input is that it is related to the spike train statistics of the driven cell. For a statistically homogeneous network this can be found (in theory) from a self-consistency condition connecting the correlation function of the input noise to the correlation function of the neuron under consideration. This has been investigated for a network of rate units, e.g. in the famous paper by Sompolinsky, Crisanti, and Sommer Phys. Rev. Lett. 1988, or for spiking neurons by Lerchner et al. Neural Comput. (2006). The correlation times caused by the nonlinear interactions in the network can easily become larger than those induced by the synaptic filter, see, for instance, Wieland et al Phys. Rev. E (2015), specifically, if synaptic amplitudes in the network are large (Ostojic, Nat Neurosci 2014).

It would be good if the authors had a look at these papers and commented whether network-interaction- induced correlations may also play a role in the considered system. One indication for long correlation times in the background noise (before the synaptic filter stage!) would be that a substantial fraction of neurons displays a coefficient of variation (CV) of the interspike interval that is larger than one. Put differently, if the spike train statistics of the neurons that generate the background noise is Poisson-like or even more regular (CV equal one or smaller than one, respectively), one can exclude that the network noise contributes significantly to the color of the background noise. The latter would then be indeed dominated by the synaptic filter as assumed by the authors.

We fully acknowledge that recurrent networks can generate correlated presynaptic activity, producing additional temporal slowing beyond the synaptic filter time constant. As explained on page 2 of this document, there is reason to believe that such effects play little to no role for To the best of our knowledge, we performed the only recordings of the correlation structure in this network. Judging by this evidence, there is very little room for additional sources of correlation beyond the synaptic filter time constant. Obviously, in vivo, the conditions might be different. However, in the absence of any quantification of the degree of correlation in vivo, there is no scientifically sound way to estimate the impact of this effect on our results.
We acknowledge the limitations of Gaussian statistics in modelling in-vivo input correlations in the manuscript:

> There is no guarantee that input correlations in vivo follow the exact same pattern. Although the PSC decay time constants represent a lower bound for the input current correlation time, multiple processes may prolong it. Our choice of 10 ms slightly exceeds the 7 ms we observed in L4 network reverberations. This experimental design thereby accommodates, to some degree, possibly slower correlations in vivo.

This said, we would like to point out that, should longer correlation times occur in vivo, those would, according to theoretical predictions, further *improve the response of the spiny stellates* - beyond the performance that we measured here. How this beneficial effect compares to the detrimental impact of a potentially stronger background fluctuation is impossible to know. It will have to be measured.

2) On p.2 the authors write:
"In cortical networks, the postsynaptic effect of local, recurrent inputs reflects a balance of inhibition and excitation, and it is largely asynchronous from neuron to neuron."

This might be true but the weak remaining cross-correlations can have a strong impact on the population activity (see the famous contribution on this problem by the Bialek group, Schneidman et al. Nature 2006). So it would be important to state how strongly the L4 neurons are correlated exactly. And one could take into account how weakly correlated noise sources to different L4 neurons could mimic this effect. At least this problem should be mentioned and the limitations of the current approach (based solely on single-cell activity) should be pointed out. To our knowledge, information on the correlation between L4 spiny stellates are not yet reported. We now mention the limitation of the current approach:"

"Because in vivo a thalamocortical volley is immediately followed by strong feedforward and feedback inhibition 37,38, our data indicate that the slower correlation time of the noise is essential for the fast encoding that is actually achieved by L4. Note that in spiny stellate cells in vivo, the background noise largely reflects recurrent activity, and the histogram bars after the initial response might be modified as a consequence of the thalamocortical EPSC. However, only the first one or two bars are relevant for our conclusion (Fig. 5D)."

I do not see how the authors have discussed or even only mentioned the contribution of cross correlations among the neurons (not to be confused with the temporal correlations of the noise or the spike train) for the considered problem. If neurons are not completely independent of each other, i) the population rate would be much more noisy than for independent neurons and ii) also the presynaptic input would be shaped by this. This may affect some of the arguments made in the context of fine tuning of population size, cell size, and correlation time of the noise. The authors' approach assumes complete independence (a perfectly asynchronous state); please, comment on the limitations of these conclusions due to possible violations of this assumption.

We have addressed the possible correlations by stating that, to the best of our knowledge, nothing has been published about the correlation between L4 stellates. The only way to address this is through speculation. This is an extension of the previous comment, and we refer to our answers there.

3) Statements about the DC, AC, and noise injections should be completed with a table of the used values.

The authors write on p.15

"We adjusted the input's standard deviation, σI, based on the input resistance of the neuron, to assure that the voltage fluctuations were similar in all neurons. Along with σI, we also scaled the amplitude of the sinusoidal component Asin=0.37·σI. A constant, direct current (DC) 〈〉 was added and adjusted to maintain a target firing rate of ~5 Hz."

This is not reproducable, because we do not know the SD of voltage fluctuations. Different SDs could in combination with different DC currents result in the same 5 Hz firing rate. I suggest to give all the values in a table in the supplementary material: neuron number, correlation time and SD of OUP input current, amplitude of sinusoidal component, DC current, measured mean firing rate, measured maximal amplitude of firing rate modulation. In this way the reader (and in the first place, this reviewer) would get an idea about i) the variability of cells, ii) the susceptibility of the voltage variance with respect to the noise amplitude, iii) the (non)linearity of the signal transmission.

The question of linearity is central to the dynamic gain approach, which is why we explicitly tested for linearity. Specifically, we compared the experimentally measured EPSC response with the response we predicted under the assumption of linearity. The result strongly supports the assumption of linearity for the EPSC response. This is shown in Fig. S1 “Linear ansatz to quantify encoding is confirmed by comparison of three different approaches” (previously Fig S3)

The assessment of linearity through comparison of average firing rate 〈v〉 and sine-modulation of the rate Av is also available in the manuscript, by way of the vector strength (VS). This can be appreciated from the equation in Fig S1:
〈v〉/Av =2VS.
The vector strength values for the individual trials are shown in Fig. 4 of the manuscript. We show it here next to the vector strength values for the individual trials that constitute Fig. 2:

The tables with the other requested information are now included in the Supplementary materials and reproduced here:

I am happy with the revision regarding this point. Thank you for adding the tables.

4) The authors compare the stimulation by a (comparatively) weak periodic signal and the estimation of the linear response function from the vector strength to the estimate of the response function from the full input-output cross-spectrum between the (non-weak) Gaussian noise stimulus and the output spike train. That these functions are the same (at least for an sufficiently weak cosine stimulus) is, according to my understanding, a consequence of the Furutsu-Novikov theorem that has been recently (re)derived in the context of spiking neurons by Lindner Phys. Rev. Lett. 2023. This connection should be mentioned.

Thank you for the reference. We now incorporated it in the text box on Dynamic gain:

"An alternative method to derive the dynamic gain function does not require an additional sinusoidal input (see Methods: Dynamic gain calculation and Fig. S1D to S1E) because the stochastically fluctuating input is, itself, composed of various frequency components. The results of these two approaches are not only theoretically identical (Stubenrauch and Lindner 2024) but also identical in our experiments (Fig. S1E)."

The given reference is not correct: Stubenrauch and Lindner derived a Furutsu-Novikov-like theorem for Poissonian spike trains with rate modulation – this is not relevant, I think, for what is done in the submitted paper. The correct (original) theorem applies for Gaussian signals (as used by the authors); it is the one rederived in the paper by Lindner that I mentioned in my first report. I do not understand why the authors cite here the other paper.

This misunderstanding is explained by our wish to follow the reviewer's suggestion, combined with two unfortunate facts:

1) The reference given by the reviewer is incorrect; a "Lindner et al. 2023" does not exist.
2) We interpreted the explanation of the incorrectly referenced paper as pointing to "Stubenrauch and Lindner 2024" as the most likely intended reference.

Point 1) sent us searching for papers by Prof. Lindner that (re-) derive the Furutsu-Novikov as per the description "the Furutsu-Novikov theorem that has been recently (re)derived in the context of spiking neurons by Lindner Phys. Rev. Lett. 2023"
Our search turned up only one explicit rederivation of this theorem by Prof. Lindner, namely *Stubenrauch and Lindner 2024*, which is why we referenced that paper - in good faith.

Given the new information in this second review, we now assume that the reviewer's sentence does not refer to a rederivation of the Furutsu-Novikov theorem, but instead to an application of the theorem in the derivation of the Fluctuation dissipation theorem in PMID 36399734 (Lindner Phys.Rev.Lett. **2022**). We hope that this is the reference the reviewer intended us to insert. The sentence now reads

> "The results of these two approaches are not only theoretically identical (Lindner 2022) but also identical in our experiments (Fig. S1E)."

5) Why the cell with larger dendrite has a broader bandwidth of transmission has been discussed at length in the paper by Ostojic et al. J Neurosci (2015). The paper is already cited in the paper but the mechanism should be briefly explained because the difference in bandwidth between layer IV and V cells is such an important starting point of the paper. Additional references that might be useful and merit citation in this context are those by Gowers and Richardson Phys. Rev. Res. (2023) and Gowers, Timofeeva, and Richardson PLoS Comput. Biol. (2020) who studied analytically response functions in spatially extended neuron models.

We added a sentence and a reference to our earlier analysis of different effects of dendrites on encoding bandwidth (Zhang, C., Revah, O., Wolf, F., and Neef, A. (2024). Dynamic gain

decomposition reveals functional effects of dendrites, ion channels and input statistics in population coding.)

“These critical features include the correlation time of the background noise (Brunel et al. 2001), the dynamics of action potential generation (Brunel et al. 2001, Naundorf et al. 2005), and the dendritic size of the neurons involved (Ostojic et al. 2015, Eyal et al. 2014). Dendrites amplify the impact of rapid input fluctuations because they shunt more slowly changing inputs (Ostojic et al. 2015, Zhang et al. 2024).”

While we appreciate the additional references, those studies are more related to the encoding of dendritic inputs, and increased dendritic conductances. We felt that for the topic of our study, it would be fair not to include those references because the inputs onto the spiny stellate cell are all electrotonically close to the soma (Segev I, Friedman A, White EL, Gutnick MJ. Electrical consequences of spine dimensions in a model of a cortical spiny stellate cell completely reconstructed from serial thin sections. J Comput Neurosci. 1995;2: 117–130. doi:10.1007/BF00961883)

I understand this argument but do not see, why the authors do not share it with the readers. I recommend to briefly mention the effect of the dendritic tree on transmission bandwidth (and cite the above references) and then state why this is not relevant for the spiny stellate cells (citing the paper mentioned in the reply).

While such informative context would be of great merit in a review paper or textbook, we believe that this manuscript is not the place for such a discussion. We specifically wrote this manuscript to be accessible to a general audience. We have already had reviews by experimentalists, and their comments showed that any theory not required to understand the problem will only reduce accessibility for physiologists and other life-science experimentalists. The mission of this manuscript is to connect different branches of neuroscience so that more experimentalists employ theory-driven approaches.

Minor issues

6)p.16
"Fourier transform of the autocorrelation" This is well-known as the power spectrum!

In our theory-oriented papers, we indeed make that transition via the Wiener-Khinchin theorem. However, I asked a number of non-computational experimentalists whether they were aware that the power spectrum is, in fact, the Fourier transform of the autocorrelation. Our wording reflects our deference to the broad readership of PLoS Biology.

I do not understand why the lack of knowledge in a certain group of researchers keeps the authors from adding a simple parenthesis “(also known as power spectrum)” to mention an important term in the theory of stochastic signals. I highly recommend to add it.
Done

The complex dynamic gain function *G(f)* was calculated as the ratio of the complex conjugate of the Fourier transform of the STA, $F(STA_I)^*$, and the Fourier transform of the autocorrelation of the stimulus (its power spectrum):

Reviewer #4:

I am still convinced of the merits of this paper and support publication of a version with minor corrections.

1. The authors haven't quite addressed my question about their choice of a 3 Hz firing rate regime. They say the choice was not prompted by an established physiological value.

We stated that an established physiological value does not exist in the literature.

It would be enough to say in the paper why they chose the value. Were they seeking a particular spiking regime?
We now added the following explanation:

A constant, direct current (DC) was added and adjusted to maintain a target firing rate of ~5 Hz.

In the absence of in vivo measurements of spiny stellate cells' firing rates, we chose this value because the neurons were able to sustain it for many seconds without detectable changes in AP shape. Lower values would inversely prolong the required measurement duration and preclude the application of two different input statistics to the same neuron.

Additionally, could they sort out the apparent inconsistency between 3 Hz and 5 Hz in the text and figure caption, as also noted by another reviewer?
We apologize for our negligence! It was an oversight, which is now corrected. The figure legend now also reads "3 Hz".
Thank you for catching that!

2. It would be useful if the clarifications on what "special care was taken" in the uncaging experiments were also described in the paper, not just in the response to my comment, for the benefit of readers.

We thank the reviewer for this suggestion.

We now added to the methods section:

Specifically, we repeatedly checked that the uncaging UV focus was outside the boundary of the barrel we recorded from. Because the dendrites of L4 neurons are almost completely confined to a barrel, this largely excludes the chance of direct contact of the released glutamate with the stimulated neuron's dendrites. In coronal slices, we performed release at least 200 μm away from the recorded Layer 5 neuron soma. Successful separation of uncaging and reverberation detection was indicated by a delay of at least 25 ms from the UV pulse onset.
