# Supplementary material for: Encoding performance of cortical neurons critically depends on their morphological and neurophysiological properties
Source: PLoS Biol. 2026 May 14;24(5):e3003789. doi: 10.1371/journal.pbio.3003789 (PMC13175474; doi:10.1371/journal.pbio.3003789)
Supplement: Attachment — Submitted filename: Responses to the reviewer comments.docx [file pbio.3003789.s007.docx]

Reviews PBIOLOGY-D-25-02148R1

We want to thank the reviewers for a truly insightful review. The comments were absolutely on point and allowed us to improve our explanations. Our responses are given below in red. Quotes from the manuscript are indented. Inside the quotes, only the changes are labelled red.

# Reviewer #1

This paper introduces an interesting and important question: how neural populations can faithfully encode inputs despite background noise, and how neurophysiology impacts encoding. However, the major problem with the paper is that the populations in question never seem to interact with one another, either in the experiments or, from what I can tell from the methods, in the computational model. Therefore, what seems to be studied is a collection of independent neurons whose activity is summed to simulate a population, where "correlated noise" inputs take the place of recurrent connections in the population.

The authors would need to incorporate additional neurons in the modeled population (with explicit recurrent connections) to make this argument/analysis more convincing. The experimental results are nice. The fact that L4 neurons have poorer high-frequency tracking than L5 neurons is interesting and compelling, as is the result that slower time constants in the background noise signal let a neuron more faithfully follow high-frequency inputs. But again, it's a stretch to describe it as a population response. In conclusion, while the results, including the model, are indeed interesting and important, they don't quite show what the authors suggest, so the paper would need to either include additional experiments and simulations, or be rewritten to less ambitiously describe the implications of the results.

The reviewer is correct; both in the model and in the experimental design we infer the consequences of connectivity based on summed activity of a distinct population of neurons. We believe that for this specific population of Layer 4 neurons, our inference and conclusion is acceptable because we are examining the *response within 1 or 2 milliseconds* of the thalamocortical EPSC (see Figures 5 and S1), which is too early for any recurrent synaptic influences on the output.

We now clarify this in the results:

“Because in vivo a thalamocortical volley is immediately followed by strong feedforward and feedback inhibition 37,38, our data indicate that the slower correlation time of the noise is essential for the fast encoding that is actually achieved by L4. Note that in spiny stellate cells in vivo, the background noise largely reflects recurrent activity, and the histogram bars after the initial response might be modified as a consequence of the thalamocortical EPSC. However, only the first one or two bars are relevant for our conclusion (Fig. 5D).”

## General Comments

1. "In cortex, individual neurons fire no more than a few action potentials (APs) each second" is really only true in rodents. NHPs, for example, have healthier and more robust firing rates.

The appropriate sentence in the introduction now reads:

“In mouse cortex, individual neurons fire no more than a few action potentials (APs) each second.”

2. The introduction should spend a few more sentences explaining dynamic gain curves/analysis (introduced in lines 31-33 on page 1) to make sure that the reader understands the following statements (e.g. lines 38-42) at least superficially, without having to review the cited papers. This concept is easy to understand once one looks at the methods, but still useful to not have to flip back and forth between sections.

The editor made it possible to place the explanations in a new Text Box directly under the introduction and keep the flow of the introduction largely unchanged. The appropriate sentences now read:

“The encoding capability of a population may be inferred from the dynamic gain curves of its constituent neurons. This is a measure of a neuron’s ability to lock its spikes onto the frequency components of its input (Text Box 1, Fig. S1 A and S1 B). Thus it represents the temporal precision with which the combined firing rate of the neurons can track changes in a common input stream^6^ (see Figure S1 G and S1 H).”

3. Text states "Its amplitude was chosen to evoke irregular AP firing at a basal rate ν_0_=3 Hz (Fig. 1B)" but Figure 1A caption says: "postsynaptic neurons firing asynchronously at about 5Hz"

We corrected this. The legend now reads

“Schematics showing a simplified neural network comprised of a single presynaptic neuron connected to ‘N’ postsynaptic neurons firing asynchronously at about 5Hz”

4. "background noise" driven by a larger population of neurons than N, where N is the size of the subpopulation? (in reading the rest of the paper, I see that N is the total population and there are no other neurons, so Figure 1A should be corrected).

The labelling on Fig 1A has been appropriately corrected.

5. The model is supposed to capture the projections patterns in L4 cortical neurons, but it was unclear from the text how realistic it is that the N neurons have the same input AND the same output to the readout neuron? Wouldn't a more realistic scenario involve some variability in the connectivity between inputs and output? The question of "How salient this minimal input is for the readout neuron …" will also depend on this question. (In reading the methods, I understand that there is no output neuron, and you sum together all of the activities in time bins, etc. Again, Fig. 1A is misleading).

The cartoon in Figure 1A has been modified to reflect that not every L4 neuron that receives thalamocortical input outputs onto every L2/3 neuron. The connections from specific thalamus to layer 4 on to layer 2/3 in the rodent barrel cortex have been studied in quite some detail by Sackman’s group and others (Feldmeyer, Staiger). Although these connections are clearly very very strong, precise quantification of connectivity across **two** synapses is unknown and there must, of course, be variability. While we cannot represent reality in a model with precise quantification, we do make our point by providing “ballpark” figures that reflect the strength and rapid relay characteristics of these connections.

6. Figure 1C,D when talking about population size, does it refer exclusively to the postsynaptic population? How does that interact with the size of the non-targeted neurons?

The model represents only the neurons that receive thalamic input and relay it to a L2/3 cell (blue population in Fig. 1A). Activity of the non-targeted neurons (gray in Fig. 1A) is reflected only in the correlated background input. As explained above, the recurrent effect of a single input pulse will only be manifest after several milliseconds and will therefore not affect the quantification represented in panels D-G.

7. It would be helpful if Figure 1 had a panel with more detailed example of connections between the dendrites, inputs, etc. in order to help the reader more easily understand the relationship between parametric changes and Pdetect

The effect of dendrites, here and in all the papers that studied it, has always been associated with the electrotonic load that comes with the added membrane. We added a sentence to the introduction and in it also point to our earlier analysis of the mechanisms ():

“These critical features include the correlation time of the background noise (Brunel et al. 2001), the dynamics of action potential generation (Brunel et al. 2001, Naundorf et al. 2005), and the dendritic size of the neurons involved (Ostojic et al. 2015, Eyal et al. 2014). Dendrites amplify the impact of rapid input fluctuations because they shunt more slowly changing inputs (Ostojic et al. 2015, Zhang et al. 2024).”

“These critical features include the correlation time of the background noise (Brunel et al. 2001), the dynamics of action potential generation (Brunel et al. 2001, Naundorf et al. 2005), and the dendritic size of the neurons involved (Ostojic et al. 2015, Eyal et al. 2014). Dendrites amplify the impact of rapid input fluctuations because they shunt more slowly changing inputs (Ostojic et al. 2015, Zhang et al. 2024).”For L4 spiny stellate cells this assumption is affirmed by the serial electron microscopy study which examined the input sites of thalamocortical synapses (Segev et al. 1995)

8. Why does "Enlarging the population size (N) … reduced the fluctuations in population rate"?

Since the output of individual neurons fluctuates differently, but at the same mean rate, increasing the number of neurons averages out the individual fluctuations and leads to a more stable population rate. The quantitative statement is given in Fig 1B; the fluctuation amplitude drops with 1/sqrt(N).

9. What is "vector strength" in Figure 2G? What test was used for significance? (Again, clear in methods, needs 1 sentence description in results).

This is now explained in the new text box 1

10. In figure 4 C and E, the black bars read "10k trials." Should just be 10?

No, 10k is correct. The vast majority of trials do not evoke a spike, which explains the sparsity of spikes despite the large number of trials.

11. The aEPSC (fig 5) was injected into the same neurons every 50 ms (20 Hz) over an extended period of time. Could that cause any plasticity over the timescale of the experiment?

As can be seen in supplemental figure 1, the 20 pA injection for the EPSC is extremely small relative to the peak-to-peak current fluctuations of the background activity, and therefore a plasticity reaction to the repetitive EPSC injection is highly unlikely.

12. Figure 5 convincingly shows that inputs during background noise w/ longer correlations are more likely to elicit temporally precise responses in the recorded neurons. But the 10 ms tau condition also looks like the background noise is overall lower amplitude (in 5A). Maybe that is an illusion, but could you quantify that amplitude as well (in addition to the overall population activity, which you showed remains the same). Also, what is the relationship between the amplitude of the background noise and the likelihood that an AP will be elicited? From the data, it's hard to tell if the 2m tau condition just reduces the total number of AP elicited OR if it ends up jittering them across the population so that they "average out" across time?

The details of the current design are explained in the method section

“We adjusted the input’s standard deviation, σI, based on the input resistance of the neuron, to assure that the voltage fluctuations were similar in all neurons. ”

The voltage fluctuations resulting from 10 ms correlated input might *appear* to have lower amplitude because the 50 ms sections are too short to give a realistic impression of the peak-to-peak fluctuation amplitude of the slower fluctuations. The following graph illustrates this very well. It shows, over a longer time interval, the responses to four trials (two with 2 ms correlation time - orange, two with 10 ms correlation time - green). Looking at any one 50 ms interval, the 10 ms fluctuations might appear to have lower amplitude, but looking over a few 100 ms, it becomes clear that this is just an effect of the limited interval duration.

13. For Figure 5, you state that: "In these experiments, since the noise is uncorrelated across sweeps, each sweep may be considered the equivalent of a different L4 neuron in response to the same aEPSC." but that's not quite right, because the response of the neurons in a population are really going to be impacted by circuit-level activity in response to the stimulus, which isn't fully captured by the background noise correlations you are inputing. For example, in the model you developed to study this question, does the activation of just one neuron propagate through the network? In a real network, one excited neuron might end up inhibiting others in its cohort, possibly even others that receive direct input from the presynaptic neurons. So while the experiment correctly simulates a large population of independently activated neurons, it likely fails to capture the interactions that would be experienced in a full network. I realize that restricting the analysis to the 2ms following the input reduces/obliterates the impact of the network on the single-neuron responses, but what it wouldn't reduce is the impact on a downstream decoder whose job it is to interpret the patterns of inputs from that population.

As described in results and methods, our detection probability (graph 5D) reports the probability that the population response *within the 1 and 2ms windows* exceeds the 95th quantile of the pre-stimulus response distribution. To simulate that response, the model does not require recurrencies.

In the discussion, we had referred to the impact of the network inputs:

“Strong feedforward and feedback inhibition leaves only a narrow window of about 2 ms during which these cells must respond (Gutnisky et al. 2017, Simons and Carvell 1989, Gabernet et al. 2005) ”

The strong feedforward inhibition most likely truncates the response in vivo. The resulting lull in the population response might simplify detection by downstream neurons, but that is a speculation.

The sentence that we added in the results section clarifies this:

“Because in vivo a thalamocortical volley is immediately followed by strong feedforward and feedback inhibition 37,38, our data indicate that the slower correlation time of the noise is essential for the fast encoding that is actually achieved by L4. Note that in spiny stellate cells in vivo, the background noise largely reflects recurrent activity, and the histogram bars after the initial response might be modified as a consequence of the thalamocortical EPSC. However, only the first one or two bars are relevant for our conclusion (Fig. 5D).”

14. Text states that "Each neuron has two distinct sources of input: extrinsic projections and massive local, recurrent connectivity." but there is no mention of the recurrent connections in the "Theoretical model" section of the methods. Instead, it seems like the recurrent connections are not explicit in the model, but are simulated by time-varying noise inputs?

That is correct.

# Reviewer #2

The authors of the ms study the response properties of excitatory pyramidal cells in layer IV and V of the somatosensory barrel cortex in mice in vitro. They demonstrate that the larger cells in layer V have a larger frequency bandwidth than the smaller principle neurons in layer IV, i.e. they respond reliably to a sinusoidal input current with a modulation of their firing rate even when the stimulation frequency is as high as several hundred Hz. These results hold true for a short-correlated background noise (in the in-vitro experiment represented by a noise stimulus generated on a computer) but are drastically changed when a longer correlated noise (with a correlation time of 10ms) is used, for which also the cells in layer IV display a high-frequency transmission. Moreover, the authors then argue that synaptic filter times are indeed longer in layer IV than in layer V, also the number of neurons and their speed of reaching the firing threshold is adapted that even weak inputs such as a single thalamo-cortical input spike, going to a subpopulation of N cells, can reliably be detected.

The results presented in this paper, in particular the experimental ones, are remarkable and an important contribution to a problem that has been predominantly studied by theoreticians. I am in favour of publishing a suitably revised version of the ms that takes into account the questions and recommendations listed below.

## Major issues

1) The authors study the role of the correlation time of the background fluctuations, however, as the source of correlation they name only synaptic filtering. In the theoretical literature, one additional potential source of correlation are the recurrent activity itself, that, specifically at larger synaptic amplitudes, can give raise to long correlations exceeding those introduced by synaptic filter times, see e.g. Ostojic Nature Neurosci 2014. The authors make a point that in their in vitro recordings correlation times match the synaptic decay times, but they should still discuss this second (more complicated) source of correlations and argue why - or, better, under which conditions - the synaptic filtering will dominate the correlation time.

Thank you for the suggestion. We added a paragraph to the discussion:

“An alternative mechanism for slow input fluctuations could be a slow oscillation of the recurrent network itself. Although this would assure conditions for a rapid response of L4 neurons without the need for special synaptic receptors, those oscillations would pose a serious problem for L4 function because it would create prolonged periods during which most neurons were far from threshold and the population’s input sensitivity would be reduced. Indeed, in-vivo recordings from mouse S1 show that during active sensing periods, the networks in S1 switch to asynchronous activity^31^, thereby avoiding intermittent drop-outs. The input correlation time is at least as long as the decay time constant of the dominant postsynaptic currents, even if the network activity is completely asynchronous. The presence of slow synapses, thereby, allows L4 neurons to consistently respond rapidly.”

2) On p.2 the authors write:

"In cortical networks, the postsynaptic effect of local, recurrent inputs reflects a balance of inhibition and excitation, and it is largely asynchronous from neuron to neuron."

This might be true but the weak remaining cross-correlations can have a strong impact on the population activity (see the famous contribution on this problem by the Bialek group, Schneidman et al. Nature 2006). So it would be important to state how strongly the L4 neurons are correlated exactly. And one could take into account how weakly correlated noise sources to different L4 neurons could mimic this effect. At least this problem should be mentioned and the limitations of the current approach (based solely on single-cell activity) should be pointed out.

To our knowledge, information on the correlation between L4 spiny stellates are not yet reported. We now mention the limitation of the current approach:”

“Because in vivo a thalamocortical volley is immediately followed by strong feedforward and feedback inhibition 37,38, our data indicate that the slower correlation time of the noise is essential for the fast encoding that is actually achieved by L4. Note that in spiny stellate cells in vivo, the background noise largely reflects recurrent activity, and the histogram bars after the initial response might be modified as a consequence of the thalamocortical EPSC. However, only the first one or two bars are relevant for our conclusion (Fig. 5D).”

3) Statements about the DC, AC, and noise injections should be completed with a table of the used values.

The authors write on p.15

"We adjusted the input's standard deviation, σI, based on the input resistance of the neuron, to assure that the voltage fluctuations were similar in all neurons. Along with σI, we also scaled the amplitude of the sinusoidal component Asin=0.37·σI. A constant, direct current (DC) 〈〉 was added and adjusted to maintain a target firing rate of ~5 Hz."

This is not reproducable, because we do not know the SD of voltage fluctuations. Different SDs could in combination with different DC currents result in the same 5 Hz firing rate. I suggest to give all the values in a table in the supplementary material: neuron number, correlation time and SD of OUP input current, amplitude of sinusoidal component, DC current, measured mean firing rate, measured maximal amplitude of firing rate modulation. In this way the reader (and in the first place, this reviewer) would get an idea about i) the variability of cells, ii) the susceptibility of the voltage variance with respect to the noise amplitude, iii) the (non)linearity of the signal transmission.

The question of linearity is central to the dynamic gain approach, which is why we explicitly tested for linearity. Specifically, we compared the experimentally measured EPSC response with the response we predicted under the assumption of linearity. The result strongly supports the assumption of linearity for the EPSC response. This is shown in Fig. S1 “Linear ansatz to quantify encoding is confirmed by comparison of three different approaches” (previously Fig S3)

The assessment of linearity through comparison of average firing rate 〈ν〉and sine-modulation of the rate A_ν_ is also available in the manuscript, by way of the vector strength (VS). This can be appreciated from the equation in Fig S1:

〈ν〉/ A_ν_ = 2 VS .

The vector strength values for the individual trials are shown in Fig. 4 of the manuscript. We show it here next to the vector strength values for the individual trials that constitute Fig. 2:

The tables with the other requested information are now included in the Supplementary materials and reproduced here:

| **Layer 5**  Cell ID | 〈I〉  (pA) | σ_I_  (pA) | 〈𝝂〉  (Hz) |  | **Layer 4**  Cell ID | 〈I〉  (pA) | σ_I_  (pA) | 〈𝝂〉  (Hz) |
| --- | --- | --- | --- | --- | --- | --- | --- | --- |
| sl1_c1_030315 | -147.2 | 304.5 | 7.4 |  | sl1_c_250215 | 32.6 | 50.6 | 5.2 |
| sl1_c1_250215 | -32.4 | 253.2 | 5.2 |  | sl1_c2_020315 | 50.4 | 150.1 | 6.7 |
| sl1_c2_230215 | 95.3 | 354.3 | 10.2 |  | sl1_c2_030315 | 40.7 | 87.4 | 5.7 |
| sl1_c2_250215 | -28.0 | 208.0 | 5.8 |  | sl1_c3_020315 | 49.5 | 50.6 | 5.2 |
| sl1_c2_260215 | 2.3 | 121.7 | 4.7 |  | sl2_c1_020315 | 51.4 | 50.9 | 4.6 |
| sl1_c3_260215 | 2.7 | 126.6 | 4.8 |  | sl2_c1_230215 | 74.4 | 50.7 | 5.2 |
| sl2_c1_030315 | 7.1 | 162.1 | 8.3 |  | sl2_c1_260215 | 284.7 | 122.3 | 5.8 |
| sl2_c2_030315 | -36.1 | 203.2 | 5.9 |  | sl2_c2_020315 | 55.9 | 50.7 | 4.8 |
| sl3_c1_020315 | -37.8 | 152.3 | 8.1 |  | sl2_c3_020315 | 75.3 | 50.8 | 5.1 |
|  |  |  |  |  | sl3_c1_020315 | 97.2 | 51.4 | 5.0 |
|  | **-19.3** | **209.5** | **6.7** |  |  | **81.2** | **71.6** | **5.4** |

**Supplementary Table 1** Stimulus details for the recordings underlying Fig. 2. Column averages are given below the horizontal lines.

| **2ms**  Cell ID | 〈I〉  (pA) | σ_I_  (pA) | 〈𝝂〉  (Hz) |  | **10 ms**  Cell ID | 〈I〉  (pA) | σ_I_  (pA) | 〈𝝂〉  (Hz) |
| --- | --- | --- | --- | --- | --- | --- | --- | --- |
| sl1_c1_03_12_15 | 69.3 | 83.9 | 6.7 |  | sl1_c1_03_12_15 | 145.0 | 48.5 | 5.9 |
| sl1_c1_04_12_15 | 17.0 | 74.3 | 5.8 |  | sl1_c1_04_12_15 | 21.3 | 46.6 | 6.3 |
| sl1_c1_06_12_15 | -1.3 | 74.1 | 7.3 |  | sl1_c1_06_12_15 | 32.5 | 46.6 | 5.8 |
| sl1_c2_02_12_15 | -4.6 | 73.7 | 5.0 |  | sl1_c2_02_12_15 | 1.4 | 46.3 | 5.1 |
| sl1_c2_03_12_15 | 68.0 | 38.3 | 5.9 |  | sl1_c2_03_12_15 | 67.1 | 23.9 | 6.0 |
| sl1_c2_06_12_15 | 24.5 | 73.6 | 5.2 |  | sl1_c2_06_12_15 | 39.0 | 46.6 | 5.0 |
| sl1_c3_02_12_15 | 4.4 | 75.1 | 4.2 |  | sl1_c3_02_12_15 | 14.4 | 46.3 | 4.3 |
| sl1_c3_03_12_15 | 47.7 | 74.8 | 5.2 |  | sl1_c3_03_12_15 | 64.5 | 46.8 | 5.6 |
| sl1_c3_04_12_15 | 9.3 | 73.8 | 5.5 |  | sl1_c3_04_12_15 | 12.1 | 46.1 | 5.6 |
| sl1_c3_06_12_15 | 10.7 | 74.6 | 7.5 |  | sl1_c3_06_12_15 | 46.6 | 46.3 | 6.8 |
| sl1_c4_02_12_15 | 2.5 | 74.3 | 5.2 |  | sl1_c4_02_12_15 | 8.7 | 46.4 | 5.3 |
| sl1_c4_04_12_15 | -16.3 | 79.3 | 6.0 |  | sl1_c4_04_12_15 | 2.0 | 47.0 | 5.1 |
| sl1_c5_04_12_15 | 115.1 | 73.6 | 5.3 |  | sl1_c5_04_12_15 | 111.5 | 46.3 | 5.3 |
| sl1_c5_06_12_15 | 28.4 | 74.8 | 6.2 |  | sl1_c5_06_12_15 | 43.5 | 46.9 | 6.0 |
| sl1_c6_06_12_15 | 28.9 | 74.1 | 5.7 |  | sl1_c6_06_12_15 | 63.1 | 46.4 | 5.8 |
| sl2_c1_03_12_15 | 49.7 | 74.1 | 5.0 |  | sl2_c1_03_12_15 | 61.0 | 46.5 | 6.0 |
| sl2_c1_06_12_15 | 20.6 | 74.1 | 6.5 |  | sl2_c1_06_12_15 | 39.4 | 46.8 | 6.2 |
| sl2_c2_02_12_15 | 17.3 | 73.8 | 5.3 |  | sl2_c2_02_12_15 | 20.0 | 46.7 | 4.8 |
| sl2_c2_03_12_15 | 2.8 | 74.1 | 7.1 |  | sl2_c2_03_12_15 | 16.7 | 46.6 | 6.5 |
| sl2_c2_06_12_15 | 15.8 | 73.8 | 5.5 |  | sl2_c2_06_12_15 | 24.6 | 46.7 | 6.0 |
| sl2_c3_03_12_15 | 20.7 | 73.7 | 5.6 |  | sl2_c3_03_12_15 | 30.1 | 46.8 | 6.0 |
| sl2_c3_06_12_15 | 12.9 | 36.8 | 5.7 |  | sl2_c3_06_12_15 | 15.0 | 23.3 | 6.0 |
| sl2_c4_02_12_15 | 43.0 | 73.9 | 4.0 |  | sl2_c4_02_12_15 | 54.2 | 46.7 | 5.2 |
| sl2_c4_03_12_15 | 12.5 | 73.8 | 5.5 |  | sl2_c4_03_12_15 | 41.5 | 47.5 | 5.9 |
| sl2_c4_06_12_15 | 26.2 | 37.3 | 5.1 |  | sl2_c4_06_12_15 | 36.5 | 23.6 | 5.4 |
| sl2_c5_02_12_15 | 55.2 | 73.8 | 4.5 |  | sl2_c5_02_12_15 | 67.9 | 46.3 | 5.0 |
| sl2_c5_03_12_15 | -38.5 | 82.6 | 6.6 |  | sl2_c5_03_12_15 | 48.8 | 46.5 | 6.2 |
| sl2_c5_06_12_15 | -2.6 | 74.0 | 6.1 |  | sl2_c5_06_12_15 | 9.6 | 46.5 | 5.5 |
| sl2_c6_03_12_15 | 48.4 | 74.2 | 5.4 |  | sl2_c6_03_12_15 | 64.6 | 47.4 | 5.8 |
| sl2_c7_06_12_15 | -10.6 | 73.8 | 7.0 |  | sl2_c6_06_12_15 | -5.9 | 46.2 | 6.2 |
| sl3_c2_03_12_15 | 41.9 | 37.4 | 4.2 |  | sl3_c2_03_12_15 | 46.1 | 25.3 | 5.0 |
|  | **23.2** | **70.1** | **5.7** |  |  | **40.1** | **43.8** | **5.7** |

**Supplementary Table 2** Stimulus details for the recordings underlying Fig. 4. Column averages are given below the horizontal lines.

4) The authors compare the stimulation by a (comparatively) weak periodic signal and the estimation of the linear response function from the vector strength to the estimate of the response function from the full input-output cross-spectrum between the (non-weak) Gaussian noise stimulus and the output spike train. That these functions are the same (at least for an sufficiently weak cosine stimulus) is, according to my understanding, a consequence of the Furutsu-Novikov theorem that has been recently (re)derived in the context of spiking neurons by Lindner Phys. Rev. Lett. 2023. This connection should be mentioned.

Thank you for the reference. We now incorporated it in the text box on Dynamic gain:

“An alternative method to derive the dynamic gain function does not require an additional sinusoidal input (see Methods: Dynamic gain calculation and Fig. S1D to S1E) because the stochastically fluctuating input is, itself, composed of various frequency components. The results of these two approaches are not only theoretically identical (Stubenrauch and Lindner 2024) but also identical in our experiments (Fig. S1E).”

5) Why the cell with larger dendrite has a braoder bandwidth of transmission has been discussed at length in the paper by Ostojic et al. J Neurosci (2015). The paper is already cited in the paper but the mechanism should be briefly explained because the difference in bandwidth between layer IV and V cells is such an important starting point of the paper. Additional references that might be useful and merit citation in this context are those by Gowers and Richardson Phys. Rev. Res. (2023) and Gowers, Timofeeva, and Richardson PLoS Comput. Biol. (2020) who studied analytically response functions in spatially extended neuron models.

We added a sentence and a reference to our earlier analysis of different effects of dendrites on encoding bandwidth (Zhang, C., Revah, O., Wolf, F., and Neef, A. (2024). [Dynamic gain decomposition reveals functional effects of dendrites, ion channels and input statistics in population coding.](https://doi.org/10.1523/JNEUROSCI.0799-23.2023))

“These critical features include the correlation time of the background noise (Brunel et al. 2001), the dynamics of action potential generation (Brunel et al. 2001, Naundorf et al. 2005), and the dendritic size of the neurons involved (Ostojic et al. 2015, Eyal et al. 2014). Dendrites amplify the impact of rapid input fluctuations because they shunt more slowly changing inputs (Ostojic et al. 2015, Zhang et al. 2024).”

While we appreciate the additional references, those studies are more related to the encoding of *dendritic* inputs, and increased dendritic conductances. We felt that for the topic of our study, it would be fair not to include those references because the inputs onto the spiny stellate cell are all electrotonically close to the soma (Segev I, Friedman A, White EL, Gutnick MJ. Electrical consequences of spine dimensions in a model of a cortical spiny stellate cell completely reconstructed from serial thin sections. J Comput Neurosci. 1995;2: 117–130. doi:[10.1007/BF00961883](https://doi.org/10.1007/BF00961883))

## Minor issues

1)

Bad line break on lines 11/12:

The effect of backg

round synaptic noise correlation time on pDetect and on dynamic gain.

Thank you, we corrected that

2) p.3

The number of postsynaptic neurons is in the caption of fig.1 referred to as N but later in the paper, in the caption of fig.5, by n. Should it not be the same letter?

Thank you! corrected

2) p.4

The authors write:

"... the background synaptic noise in a neuron reflects the relatively uncorrelated

synaptic bombardment by excitatory and inhibitory local inputs ..."

where it is not clear whether 'relatively uncorrelated' is meant with respect to time or with respect to presynaptic neurons; I suspect (and would agree) with the latter interpretation - the authors should here use an unambiguous phrasing.

We changed the wording to

“During ongoing cortical activity, the background synaptic noise in a neuron reflects ~~the relatively uncorrelated~~ synaptic bombardment by asynchronous excitatory and inhibitory local inputs ”

3) P.6

"To this end, embedded a single, time-locked artificial EPSC (aEPSC) in the random noise ..."

-

"To this end, we embedded a single, time-locked artificial EPSC (aEPSC) in the random noise ..."

Thank you!

4) The ms ends on p.9 with two words, i.e. an unfinished sentence:

"Our findings"

Thank you!

5)p.15

APs were detected as crossings of zero membrane voltage.

-

APs were detected as upward crossings of zero membrane voltage.

(you do not want to count downward crossings as an AP time instant)

Thank you!

6)p.16

"Fourier transform of the autocorrelation"

This is well-known as the power spectrum!

In our theory-oriented papers, we indeed make that transition via the Wiener-Khinchin theorem. However, I asked a number of non-computational experimentalists whether they were aware that the power spectrum is, in fact, the Fourier transform of the autocorrelation. Our wording reflects our deference to the broad readership of PLoS Biology.

# Reviewer #3

This manuscript by Revah et al. investigates how the temporal fidelity of cortical population coding arises from specific cellular and synaptic properties. Using dynamic gain analysis, the frequency-dependent linear response of a neuron or population to input fluctuations, the authors combine theoretical modeling with electrophysiological experiments in rodent barrel cortex. They focus on Layer 4 (L4) spiny stellate cells as a “stable, defined population” receiving known thalamic input. The study tests predictions that a population’s encoding speed depends on cell number, cell size, and background noise correlation time Key findings are that L4 neurons indeed exhibit a broad high-frequency bandwidth and high response fidelity, enabled by their small size and unusually long synaptic noise correlation time. These factors appear “fine-tuned” together with the number of L4 neurons such that even a single thalamocortical spike in the input can be reliably detected in the L4 population output. Additionally, pharmacological manipulation of K_V7 (M-type) potassium channels shows that M-current activity modulates the speed of action potential initiation, thereby adjusting the dynamic gain. This suggests that the fast relay function of L4 can be gated by cholinergic (muscarinic) brain states. Overall, the authors provide a mechanistic link between cellular physiology (e.g. spike initiation kinetics, synaptic kinetics) and the sub-millisecond precision of population coding in cortex. The questions addressed are highly significant for understanding cortical information processing. While it has been known that cortical neurons can encode surprisingly high-frequency input changes, this work pinpoints how specific biophysical parameters are matched to achieve near-optimal encoding in an actual cortical microcircuit.

While the study is compelling, a few weaknesses or points of clarification should be addressed:

1)	A potential concern is how well the slice experiment results translate to the intact, awake brain. In vivo, cortical neurons receive continuous barrage from ongoing activity, and neuromodulatory tone differs across brain states. The authors should clarify the assumptions made when extrapolating from their in vitro “stable population” to in vivo function. For instance, if the slice was in an artificial down-state (with very low spontaneous activity except what was injected), how might an up-state or aroused condition (with higher baseline synaptic noise) affect the dynamic gain? They partially address this by discussing muscarinic modulation, but it would strengthen the paper to explicitly note that in awake conditions L4 might already be in a high-conductance state – and to speculate how that interacts with their findings. In particular, would the “single thalamic spike → output” rule still hold amidst ongoing cortical activity? If possible, the authors could discuss evidence from in vivo studies (e.g. Bruno & Sakmann 2006 showing reliable L4 spiking to single thalamic events) to support that their slice results reflect real function.

Of course, it is difficult to infer activity in an intact, behaving animal from data collected in an ex vivo slice. While we can speak to the encoding potential of layer 4 neurons, we are very cautious about speculating on specific behavioral conditions. Where it has been studied in vivo (e.g. Poulet and Petersen, 2008; Figure 2), background activity has been shown to desynchronize substantially during active whisker function. When the network desynchronizes, the input correlations are mainly determined by the decay time of the synaptic inputs. This is precisely the case that we study here.

2)	The claim that even one thalamocortical spike is reflected in population output is exciting but could be interpreted in different ways. Does it mean that one presynaptic spike in one VB thalamic neuron can trigger at least one spike in L4 (across the population) on essentially every occurrence? This would imply an extremely high sensitivity. Classical studies suggest thalamic inputs are weak individually, and a few synchronous thalamic spikes are needed to drive an L4 cell to fire. The authors should clarify this point. It may be that within a barrel, because there are ~100 spiny stellates receiving partially redundant thalamic afferents, the population as a whole fires at least one spike for each thalamic spike (even if any given single neuron is probabilistic). If that is the interpretation, the authors might explicitly state it to avoid confusion. As a suggestion, providing a quantitative estimate of L4 population spike probability per thalamic input (and how that compares to chance) would help substantiate the “reliable relay” claim. If this is based on modeling rather than direct measurement, that should be stated. In any case, a clearer explanation would strengthen the reader’s confidence that this is not an overstatement.

Our study does not suggest that a single thalamocortical AP will reliably drive a given L4 cell to spiking. Rather, we show that a single thalamocortical APs at the input to the L4 population will influence the spike times of its constituent neurons to be reflected reliably in the collective output.

The difference between these two cases is reflected in our experiments. Fig. 4 shows 2 x 200 trials à 50 ms (200 for each correlation time), and only around 25% of trials contain an AP, and often this AP occurs not particularly close to the preceding EPSC. The additional graph below illustrates this in a similar way, but shows longer intervals

Four responses to current stimulations with correlation times of 2 ms (orange) or 10 ms

(green). Artificial EPSCs of 20 pA amplitude were injected at the indicated time points.

The 20 pA we used as thalamocortical EPSC amplitude in spiny stellate cells is slightly below the average thalamocortical input at -60 mV. The average was reported to be 28.8 pA (Gil, Z., Connors, B.W., and Amitai, Y. (1999)), reference 33 in our manuscript.

3)	The finding that L4’s background noise has a longer correlation time than other layers is intriguing. The manuscript would benefit from a more detailed discussion of why this is the case biophysically. Do L4 cells have different receptor compositions or network connectivity that prolong synaptic events? For example, is it due to a higher NMDA/AMPA ratio, or more GABA_B involvement, or simply that most “noise” in L4 comes from recurrent activity with longer integrative times? Citing specific data (perhaps from their recordings of synaptic kinetics, or known receptor differences) would support this point. In the text, they mention “synaptic receptor dynamics” as the cause. It would help readers if the authors specify which receptors dominate L4 noise, e.g. “slow NMDA-mediated EPSPs and GABAergic currents lengthen the autocorrelation of L4 synaptic input.” This also ties into literature: slow glutamate receptors can sustain depolarizations and thus increase the integration window. A brief comparison to layers 2/3 or 5 (which likely have faster AMPA-dominated transients) could contextualize the uniqueness of L4.

You are correct to speculate that the receptors are different. We mention this in the discussion (4th paragraph)

“This difference between L4 and other layers may be explained by previous evidence that, although fast AMPA-receptors are the predominant mediators of excitatory synaptic activity in all other cortical layers, in L4 of the mouse barrel cortex, a slower NMDA-receptor with minimal Mg^2+^-sensitivity (NR2C) is mainly responsible for ongoing synaptic activity, even at resting potential^25,50^. Because encoding capacity critically depends on background correlation time (Fig. 4), the unique synaptic physiology in L4 is crucial to its function. “

To our knowledge, no other cortical neuron expresses NR2C receptor subunits. As your comment hints, such a unique physiology as a cause of the slower correlation times strengthens our point of “fine tuning”.

4)	The demonstration with K_V7 channels is convincing, but a few clarifications are warranted. K_V7

(M-type) channels are one of several subthreshold conductances (others include HCN/I_h and persistent Na^+). The authors focus on K_V7 likely because their effect was strong, but it would be useful to acknowledge whether other channels were considered or could play a role. For instance, I_h currents can also influence responsiveness by depolarizing neurons and speeding up rebound firing. Did the authors assume I_h was negligible in these L4 cells or kept constant? A sentence discussing this would show thoroughness. Additionally, XE991 (the M-channel blocker) can have off-target effects at high concentrations; the authors should note the concentration used and any steps taken to ensure changes in gain were indeed due to M-current block (e.g. reversibility, known effects on input resistance, etc.). Since the manuscript’s main claim here is that M-currents modulate spike initiation speed, they might consider directly correlating the AP onset rapidness (slope of rise to threshold) before and after M-channel block.

This would link to prior work showing that AP onset dynamics govern high-frequency encoding. Providing such a correlation (if available) would further strengthen the mechanistic point.

We pursued M-currents guided by our previous work on the critical impact of AIS ion channels in AP timing (Lazarov et al. 2018). Of course, there are numerous potassium channel types, and studying them all would be beyond the scope of this paper.

5)	The authors repeatedly use terms like “optimized” and “fine-tuned.” While they do demonstrate that L4 meets certain theoretical predictions, it would be beneficial to see how sensitive the encoding performance is to deviations in those parameters. For example, if L4 cell soma size were 20% larger, or if synaptic time constants were 50% faster, does the high-frequency cutoff drop precipitously? The manuscript would benefit from either data or discussion on this point – essentially, an analysis of robustness. Such information would convey how precisely tuned the system is and could reveal which factor is most critical. It also helps readers appreciate whether evolution had to precisely adjust all factors or if there’s some tolerance. If the authors did not quantitatively do this, they should at least qualitatively discuss it, given it’s central to their claim of fine-tuning.

The theoretical tuning curves are shown in Fig. 1. Experimentally, we wondered how L4 cells could relay TC input so quickly, given their small size, and we focused on the measured correlation times for L4 and L5 neurons.

We feel that the wording “fine-tuned” is appropriate because the tuning reflects the convergence of three completely unrelated variables: dendrite size, synaptic receptor dynamics and ion channel complement at the axon initial segment and soma.

6)	In the Discussion, the authors should amplify the implications of their findings for sensory processing and behavior. They touch on this (e.g. mentioning that coding in L4 may vary with brain state, but they could go further. For instance, the fact that L4 can reliably transmit single spikes suggests the cortex can operate on a single-spike per neuron basis for initial sensory detection. This resonates with theories of fast feedforward processing in vision and touch, where very few spikes are needed for perception. The authors might speculate on how the fine-tuning in L4 could be a general principle in other sensory cortices, or discuss if similar optimization might be found in auditory or visual L4 (noting differences, e.g. auditory cortex lacks a discrete L4 in rodents. Additionally, since they link to muscarinic control, they could mention potential behavioral scenarios: during attentive whisking, cholinergic tone is high, which might transiently enhance high-frequency transmission in barrel cortex, possibly improving detection of rapid whisker vibrations or changes in texture. Making these connections would strengthen the significance of the work for a broad neuroscience audience.

Thank you very much for the appreciation and the encouragement! We refrained from most speculations, but amended the discussion with a final paragraph:

“It is widely agreed that the purely sensory, whisker-specific input to spiny stellate cells establishes L4 as the key entrance for sensory input to cortex. Our characterization extends this view. We found that three parameters from completely different domains, i.e., population size, neuron morphology, and NMDA receptor kinetics, act together to set the observed sensitivity of L4 excitatory neurons. This allows L4 to relay the thalamic input rapidly and amplify it. At the same time, our preliminary characterization of a possible cholinergic modulation suggests that L4 may even serve as a gate, operated by neuromodulation. This exciting possibility inspires further characterization of cholinergic modulation ex vivo and, importantly, in vivo, to identify behaviorally relevant effects of cholinergic modulation on sensory performance, particularly for weak tactile stimuli, when amplification by L4 might be particularly important.”

# Reviewer #4

This is a very nice paper tying together the biophysical properties of individual neurons with a population's capacity to respond to inputs and transfer information.

My own expertise is in experimental measurement rather than computational analysis, and this is reflected in my questions/issues. They are all relatively minor.

1.	What led the authors to choose 3 Hz as basal rate in their simulations (and 5 Hz in their experimental noise injections)? Was this picked as a physiologically realistic value for L4 spiny stellates? Please indicate if so.

While several studies report firing of L4 spiny stellates under anesthesia to our knowledge only the Petersen lab has recorded from identified spiny stellates in awake animals. Crouchet and Petersen 2006 ([Correlating whisker behavior with membrane potential in barrel cortex of awake mice](https://doi.org/10.1038/nn1690)) show that at rest (no whisking), the firing rate can reach several Hz while responses are lower during whisking. The majority of in vivo studies report population (multi-unit) responses (e.g. the labs of Mathew Diamond and Miguel Nicholelis). We therefore cannot claim that the 3 or 4.5 Hz that we used constitutes a well-established physiological value.

2.	In figures 2G and 4G, the statistical testing seems to rely on a battery of pairwise comparisons with no correction for repeated testing. I realize that some of the p-values in 4G are highly significant, but what is the rationale for not implementing a more robust approach correcting for multiple comparisons?

We refrained from a correction for multiple comparisons because the correction is not as clear-cut as it appears at first glance: the values at individual frequencies might or might not be statistically independent. A thought experiment will illustrate the conundrum: If we had measured at 199, 200, 201, 202, 203 and 204 Hz, we would not expect these values to be independent of each other, yet naive correction would demand lowering the p-values. This thought experiment might sound irrelevant; however, it is borne out in Fig. 4E, where we do precisely this: we calculate the dynamic gain for each Fourier component with 1 Hz spacing. Afterwards, we apply a filter. The individual values are definitely not independent, nor is it sensible to correct for the comparison in 1000 spectral components.

Because of possible dependencies among data points and the unclear correction method for 4E, we decided not to apply a correction to 4G. As you pointed out, we hoped that this would not diminish readers’ trust in the results, because the very highly significant p-values would have survived any correction for multiple comparisons.

3.	How did the glutamate uncaging experiments ensure that there was no direct "contact" with the cell under study? It is said that "special care was taken" but which steps ensured this are not clear.

Release location and temporal delay between release and response provide strong arguments against a direct impact.

When studying L4 networks in tangential slices, we repeatedly confirmed that the uncaging UV focus was outside the barrel boundary, which is visible in these slices. Because the dendrites of L4 neurons are almost wholly confined to the barrel, this excludes direct contact of the released Glutamate with L4 dendrites.
In coronal slices, the dendrite range is not directly visible, so we resorted to using release sites at least 200 µm away from the soma we recorded from.

In all cases, we used only recordings in which the population response onset was delayed by at least 25 ms relative to the onset of the 50 ms UV pulse.

4.	How does dendrite length produce its effect on dynamic gain bandwidth? The theoretical papers looking at this issue suggested impedance load as a key factor, and intuitively one would expect input resistance to play a major role. And in the results from the experimental L5 vs L4 comparison results it is hard to disentangle dendrite length from Rin. Can the authors comment on this? There is essentially no reference to this in the paper.

The papers we cite demonstrate that larger dendrites increase the encoding bandwidth (Ostojic et al. 2015 [Neuronal Morphology Generates High-Frequency Firing Resonance | Journal of Neuroscience](https://doi.org/10.1523/JNEUROSCI.3924-14.2015);

Eyal et al. 2014 [Dendrites Impact the Encoding Capabilities of the Axon | Journal of Neuroscience](https://doi.org/10.1523/JNEUROSCI.5431-13.2014).)

They also attribute this to impedance load and the resulting attenuation of low-frequency inputs, which in turn increases the contribution of high-frequency inputs to AP initiation.

Our earlier analysis of this matter suggests that passive (morphology, impedance) aspects are typically the leading factor, although there is a more complicated interaction between the dynamics of the AP generator and the impedance load (Zhang, C., Revah, O., Wolf, F., and Neef, A. (2024). [Dynamic gain decomposition reveals functional effects of dendrites, ion channels and input statistics in population coding.](https://doi.org/10.1523/JNEUROSCI.0799-23.2023))

To keep this accessible to the reader, we clarify the leading factor and put all references in one place in the paper:

“These critical features include the correlation time of the background noise (Brunel et al. 2001), the dynamics of action potential generation (Brunel et al. 2001, Naundorf et al. 2005), and the dendritic size of the neurons involved (Ostojic et al. 2015, Eyal et al. 2014). Dendrites amplify the impact of rapid input fluctuations because they shunt more slowly changing inputs (Ostojic et al. 2015, Zhang et al. 2024).”

5.	What is the linear fit in Fig. 3D? From its appearance and judging by the caption, it seems to be a fit to the full data set (i.e. L4 and L5). I'm not sure if this makes sense - there is no obvious reason why all the points (for different cell populations with different biophysics) should be on the same line, and the constituent data sets are too small to test this properly. More superficially, if the fit is to the full data set, why is it plotted in the color of the L5 data?

Thank you for pointing out the inconsistent coloring. It is now corrected. It is indeed a fit over the full data set. We hypothesized that EPSC decay time constant and input correlation time are directly related, independent of cell type.

6.	Fig. 2D and E: are the grey lines actual best fits or just a visual guide added by hand? (and this also applies to Fig. 4C and D)

The lines are fits. However, they serve merely as illustrations, not quantifications. That is why we did not include the fact that these are actual fits. This was a deliberate decision to avoid the potential misconception that we used the fit amplitude to calculate the vector strength. Early studies used this method (Boucsein et al. 2009, Köndgen et al. 2008) but it proved less reliable than the method used here.

7.	P. 8, lines 37-38: "further demonstrate that … EPSC decay time constants". I'm not sure the authors have fully demonstrated this. "Our findings indicate", perhaps?

We hedged the statement:

“the slow kinetics of the noise is very likely due to long EPSC decay time constants”

8.	P. 9, line 23: The discussion section seems to stop in mid-flow. Has part of it gone missing?

Thank you for pointing this out. The left-over stub has been removed.
